# Predicting physiological aging rates from a range of quantitative traits using machine learning

**DOI:** 10.18632/aging.203660

**Published:** 2021-10-29

**Authors:** Eric D. Sun, Yong Qian, Richard Oppong, Thomas J. Butler, Jesse Zhao, Brian H. Chen, Toshiko Tanaka, Jian Kang, Carlo Sidore, Francesco Cucca, Stefania Bandinelli, Gonçalo R. Abecasis, Myriam Gorospe, Luigi Ferrucci, David Schlessinger, Ilya Goldberg, Jun Ding

**Affiliations:** 1 Longitudinal Studies Section, Translational Gerontology Branch, National Institute on Aging, Baltimore, MD 21224, USA; 2 Department of Epidemiology, The Herbert Wertheim School of Public Health and Human Longevity Science, UC San Diego, La Jolla, CA 92093, USA; 3 Department of Biostatistics, University of Michigan, Ann Arbor, MI 48109, USA; 4 Istituto di Ricerca Genetica e Biomedica, Consiglio Nazionale delle Ricerche, Monserrato, Italy; 5 Geriatric Unit, Azienda Sanitaria di Firenze, Florence, Italy; 6 Laboratory of Genetics and Genomics, National Institute on Aging, Baltimore, MD 21224, USA; 7 ViQi, Inc., Santa Barbara, CA 93111, USA

**Keywords:** physiological aging rate, quantitative trait, machine learning, aging clock, mortality, personalized medicine

## Abstract

It is widely thought that individuals age at different rates. A method that measures “physiological age” or physiological aging rate independent of chronological age could therefore help elucidate mechanisms of aging and inform an individual’s risk of morbidity and mortality. Here we present machine learning frameworks for inferring individual physiological age from a broad range of biochemical and physiological traits including blood phenotypes (e.g., high-density lipoprotein), cardiovascular functions (e.g., pulse wave velocity) and psychological traits (e.g., neuroticism) as main groups in two population cohorts SardiNIA (~6,100 participants) and InCHIANTI (~1,400 participants). The inferred physiological age was highly correlated with chronological age (R2 > 0.8). We further defined an individual’s physiological aging rate (PAR) as the ratio of the predicted physiological age to the chronological age. Notably, PAR was a significant predictor of survival, indicating an effect of aging rate on mortality. Our trait-based PAR was correlated with DNA methylation-based epigenetic aging score (r = 0.6), suggesting that both scores capture a common aging process. PAR was also substantially heritable (h2~0.3), and a subsequent genome-wide association study of PAR identified significant associations with two genetic loci, one of which is implicated in telomerase activity. Our findings support PAR as a proxy for an underlying whole-body aging mechanism. PAR may thus be useful to evaluate the efficacy of treatments that target aging-related deficits and controllable epidemiological factors.

## INTRODUCTION

The study of possible intrinsic aging rates is itself venerable and continues to be motivated by Peter Medawar’s pioneering question: does aging occur by a fundamental process independent of—though affected by—overt disease? [1]. To answer this question, the investigation of human aging rates must confront the experimental hurdle presented by our long lifespans. As a result, longitudinal studies of aging in human cohorts typically require significant resources invested over many years. A reproducible framework for estimating physiological age as a measurement of intrinsic age progression would permit the analysis and evaluation of treatments that target aging-related debilitation without a need for expensive long-term studies [2–4]. To be useful, biological aging rate measurements should be relatively stable across time in longitudinal studies [3, 5, 6], account for aging at different biological levels [7–9], and ideally be associated with disease risk and mortality [3, 8]. Early attempts to estimate “biological age” using individual biomarkers of aging (e.g., telomere length, pulse wave velocity, grip strength, etc) produced only modest correlations with chronological age [10–17]. Since there is little variation in human age at natural death [18], true biological age is unlikely to deviate markedly from chronological age—hence the need for a more tightly correlated measure of biological age.

Measurements of biological age based on DNA methylation patterns have provided significantly higher correlation (R^2^ > 0.8) with chronological age [19, 20] and can also be associated with disease and death [21–27]. However, the role of DNA methylation in the aging process is still largely unclear [28]; it does not account for higher biological levels such as physiology and phenotype, which are presumably affected by other aging-related mechanisms.

Given that aging is a complex process that manifests in virtually all tissues and organs [29–31], it seems likely that composite indices that summarize many molecular and physiological traits to estimate biological age may provide a route to greater understanding [5, 9]. Many efforts have turned to quantitative models for measuring biological age using sets of biomarkers; these models have had success in measuring variations associated with physiological decline, but may require certain assumptions about biological aging or carefully curated sets of traits [32–39]. An increasingly popular approach for robust measurement of biological age is machine learning, which involves building mathematical models on a set of training data to predict a target variable [40]. Machine learning has been used to predict biological age from DNA methylation patterns [19, 20], blood serum markers [41], image data [42–44], and transcriptomic and proteomic signatures [45–47]. Despite these advances, few biological age measurements integrate traits from distinct hierarchical levels (e.g., proteome, metabolome, and phenome) and across the multiple organ systems in which aging is manifest.

Here we use a machine learning approach with a broad range of biochemical and physiological traits including blood phenotypes (e.g., high-density lipoprotein), cardiovascular functions (e.g., pulse wave velocity) and psychological traits (e.g., neuroticism) as main groups from the SardiNIA longitudinal study of aging [48, 49] to estimate human physiological age, a metric for phenotypic and functional age progression [7]. From the estimated physiological age, we derive a physiological aging rate (PAR) as a measure of individual age progression relative to chronological age. This framework is used to determine the most age-responsive, well-correlated, and relevant traits with respect to physiological aging. Independently, we develop a model for estimating physiological age in the InCHIANTI longitudinal study [50, 51], for which we find results similar to those in the SardiNIA study and show positive correlation between PAR and the epigenetic aging rate (EAR) based on DNA methylation. We determine that PAR is a significant predictor of survival and is appreciably heritable. Additionally, a genome-wide association study (GWAS) reveals single-nucleotide polymorphisms (SNPs) that are significantly associated with PAR. These findings may help to elucidate the physiological and genetic underpinnings of the human aging rate.

## MATERIALS AND METHODS

Our primary goal was to predict an individual’s physiological aging rate (PAR) based on his/her quantitative traits by using machine learning methods. We investigated several machine learning models, trait selection and dimensionality reduction methods, and data cleaning and sampling strategies to develop an optimized framework. Each machine learning model was trained on the entire trait set using chronological age as the target variable. The predictive frameworks were evaluated using the coefficient of determination (R^2^) between the predicted age and chronological age. The most promising model was then used to generate physiological ages for individuals in SardiNIA and separately trained to generate physiological ages for individuals in InCHIANTI.

The general pipeline for estimating individual physiological aging rate is listed below (also see Supplementary Figure 1; we discuss the details in each sub-section of Materials and Methods):

1. The datasets were cleaned to remove missing values. The strategy for data cleaning was heuristically determined to preserve the maximum number of traits and samples (see Supplementary Materials).
2. Individuals were binned by chronological age (see Supplementary Figure 2 and Supplementary Materials for details).
3. Samples were split randomly into the training (90%) and testing (10%) sets in each of the chronological age bins.
4. Traits were selected by the Fisher score computed on the training set and trait values were transformed with linear discriminant analysis. The number of traits selected was predetermined as the number of Fisher-rank-ordered traits corresponding to the maximum average R^2^ value between predicted and actual age across 10,000 training-testing splits.
5. Machine learning models were trained to predict chronological age on the training data set and validated using the testing data set.
6. Steps 3–5 were reiterated 10,000 times to create enough training-testing splits to ensure wide coverage of all participants. The physiological age of an individual was the mean predicted age across these 10,000 training-testing splits.
7. The physiological aging rate (PAR) was calculated as the ratio between physiological age and chronological age for a given individual.

For the SardiNIA study, after computing PARs, we estimated the heritability of PAR from pedigree information. We also treated PAR as a quantitative trait to perform a genome-wide association study (GWAS) and to identify genetic variants (i.e., single nucleotide polymorphisms, SNPs) significantly associated with PAR. We examined the reproducibility of PAR measurements across time using the follow-up studies from each dataset. We determined significant differences in PAR measurements between deceased and living subjects. For the InCHIANTI study, we compared our PARs with corresponding DNA methylation age measurements [19, 20].

### Datasets

#### SardiNIA longitudinal study

Funded by the National Institute on Aging in 2001, the SardiNIA Project (age range 14.0 to 101.3 years, with a mean of 43.7 years; 57% female) is a longitudinal study of human aging on the island of Sardinia, which is notable for its long-lived population [48, 49]. Several characteristics made this population ideal for modeling physiological age. First, the study cohort is relatively homogenous, which reduced genetic noise. Second, environmental factors are more controlled in an island population, which reduced possible noise due to environmental heterogeneity. Third, the SardiNIA dataset included sequencing information for most of its cohort, making the study suitable for GWAS [52]. The size of sequence coverage and availability of family relationships permitted heritability estimates from pedigree information. The longitudinal nature of the SardiNIA study (i.e., three follow-up waves in addition to the baseline study) allowed us to study individual aging rate dynamics across time [52].

The dataset included over 6100 participants and more than 200 physiological and cognitive traits in total. After cleaning missing entries in a trait-blind, iterative manner, we retained over 4000 subjects and roughly 140 traits with complete data; they were used for model development.

#### InCHIANTI longitudinal study

The InCHIANTI study was designed to understand the physiological factors that affect mobility and included measurements from many different physiological systems [50, 51]. The studied population spanned two sites: Greve in Chianti (11,709 inhabitants >65 years) and Bagno a Ripoli (Village of Antella, 4704 inhabitants >65 years). The study started in 1998 with 1,463 subjects (age range 21.0 to 102.0 years, with a mean of 69.0 years; 56% female) and included four follow-up waves with the fourth wave retaining 687 subjects. The majority (1,020) of the subjects were elderly (>65 years old). The study included approximately 2,500 trait variants spanning six main physiological subsystems: central nervous system, peripheral nervous system, perceptual system, muscles, bones/joints, and energy production/delivery [51]. The InCHIANTI study provided similar advantages as the SardiNIA study in genetic and environmental homogeneity and its longitudinal nature. In addition, InCHIANTI included data on DNA methylation for many subjects, which were used to calculate DNA methylation age estimates [50].

For the SardiNIA and InCHIANTI cohorts, we chose to apply the machine learning models to the full set of traits of each cohort. The main reason for this choice is that the vast majority of current machine learning methods require that individuals have all trait values available (i.e., no missing data), and consequently, if we were to try to use the same traits for both cohorts, we could use only the smaller list of overlapping traits. Another advantage of the current approach is that the same method can be applied to data from any new study without requiring that it provides exactly the same set of traits.

### Machine learning algorithms

We explored both classification and regression models including the random forest classifier (RFC) [53], elastic net (ElNet) regression [54], k-nearest neighbors regressor (kNNR) [55], LASSO regression [56], multiple linear regression, and support vector machine (SVM) [57] (see Supplementary Materials for model specifications). Classification models required bin labels, which were computed as the center chronological age of each bin. Regression algorithms utilized the individual chronological ages but required binning to uniformly sample subjects from the age bins. The machine learning algorithms were adapted from the open-source Scikit-Learn machine learning library [58].

#### Random forest classifier

The random forest classifier from the Scikit-Learn library [58] was selected for its optimal predictive performance. Our model used 30 estimators, the Gini impurity criterion, minimum sample splits of 2, and minimum sample leaf sizes of 1. These model parameter choices were determined heuristically. The primary advantage afforded by the random forest model is its natural ability to include ordinal and binary features that are present in both SardiNIA and InCHIANTI studies (e.g., yes/no answers to questionnaires, categorized qualitative ratings of high, moderate, and low, etc).

### Trait selection and dimensionality reduction

#### Fisher trait scoring and selection

We utilized the Fisher scoring method for trait selection [59]. For the training data, the trait values and the corresponding chronological age bin labels were used to assign a Fisher score to each trait. The number of Fisher rank-ordered traits that maximized the average R^2^ between predicted and actual ages over 10,000 training-testing splits was used for model training. After trait selection, the RFC model included 79 traits while the common-trait RFC model included 52 traits in the baseline SardiNIA study. Further dimensionality reduction was performed using linear discriminant analysis (LDA), which slightly improved the predictive accuracy of the models (see Supplementary Materials, Supplementary Figure 3).

#### Linear discriminant analysis

Linear Discriminant Analysis (LDA) was used for dimensionality reduction before machine learning. LDA is a generalization of the Fisher discriminant and transforms a set of traits into N-1 linear discriminants where N is the number of discrete classes (i.e., number of age bins). We implemented the default “LinearDiscriminantAnalysis” function from the Scikit-Learn library [58].

### Statistical metrics

To measure the predictive performance of the different predictive models, we employed several statistical metrics to determine the accuracy of model-predicted physiological age with respect to chronological age.

#### Pearson correlation ® and coefficient of determination (R^2^)

The primary measurement of correlation was the Pearson correlation *r* and the closely related coefficient of determination R^2^, which represented the proportion of the variance in the target variable that could be explained by the traits used in the model. The R^2^ value was computed as the square of the Pearson correlation coefficient *r,* (*R*^2^ = *r*^2^):

#### Mean absolute error (MAE)

The mean absolute error represented the magnitude of difference between two continuous variables. The MAE was calculated for *N* measurements of two variables (x, y) as:


$$MAE=\frac{\sum_{i=1}^{N}\left|x_{i}-y_{i}\right|}{N}$$

The MAE measured the average difference between chronological age and physiological age in a cohort of *N* individuals and is represented in units of years.

### Measurements of age progression

#### Physiological aging rate

The physiological aging rate (PAR) represented the characteristic aging rate for an individual from birth to the time of measurement and was computed as the ratio of predicted physiological age to chronological age:


$$PAR=\frac{PhysiologicalAge}{ChronologicalAge}$$

We chose to use the ratio rather than the difference between physiological and chronological age because the ratio accounts for the age of an individual such that the same difference in ages would result in a larger deviation in the PAR of a younger subject than that of an older subject.

#### Interpretation of PAR

When PAR < 1, the individual’s physiological age is less than their chronological age, they are aging slower than expected for their age cohort; when PAR > 1, the individual’s physiological age is greater than their chronological age, so they are aging faster than expected.

### Testing the association between PAR and mortality

We used two methods to study the effect of PAR on survival (or mortality) using the SardiNIA cohort in which we were able to collect information on mortality. We recorded 263 deaths among the 4,415 SardiNIA participants that had their PAR estimated using all traits. The deceased participants had ages from 16.3 to 76.7 years. We first applied the widely used survival analysis model (Cox proportional hazards model) to test whether PAR is a predictor for survival while adjusting for age and sex. The PAR measurement was base-2 logarithm transformed [i.e., log_2_(PAR)] such that the effect size of PAR on survival could be conveniently interpreted as the hazard ratio with a two-fold change of PAR. Second, we performed a comparative analysis between the 263 deceased participants and the remaining participants on their aging rates. To remove the potential confounding of chronological age, we randomly paired an age-matched living participant to each deceased participant. The differences in the PAR measurements of the pairs were then calculated as ΔPAR = PAR_deceased_ – PAR_living_, and a corresponding *p*-value was obtained from a one-sided (ΔPAR > 0) one-sample *t*-test. The age-matched comparison was performed 100,000 times and ΔPAR and *p*-values were calculated for each comparison.

### Heritability

We used POLY software (http://people.virginia.edu/~wc9c/poly) to estimate the heritability of the physiological aging rate (PAR) based on the known family structure in the SardiNIA cohort.

### GWAS

Genome-wide association studies (GWAS) have been a powerful tool to infer genetic associations with diseases and quantitative traits [60–62]. We treated PAR as a quantitative trait and employed GWAS to determine genetic loci significantly associated with PARs for the SardiNIA study.

### Trait ranking

We used three different methods to rank the traits in SardiNIA and InCHIANTI. Each method was designed to assess the relative importance of a trait to a different measure of physiological aging. First, traits were scored for their association to the physiological aging rate (PAR) using the *p*-value from a two-tailed student’s *t*-test comparing the mean trait values of individuals in the top PAR quartile and that of individuals in the bottom PAR quartile. The fold change of the trait values between the two quartiles was used to quantify the relation between significant traits and the PAR. Secondly, traits were scored for their association with the physiological age using the Pearson correlation *r* between the trait values and the measured physiological age of individuals. Finally, traits were scored for their contribution to the predictive performance (R^2^) of the model. Each trait was independently removed from the model, and the average loss in model performance over 500 training-testing splits was calculated with respect to a baseline performance of R^2^ = 0.858 (SardiNIA) and R^2^ = 0.702 (InCHIANTI). We refer to the difference in model performance after removing a given trait as the added value (R^2^) of the trait.

## RESULTS

### Intuition about physiological aging rate (PAR)

We used machine learning to infer an individual’s PAR (see Materials and Methods for details), the ratio between physiological age and chronological age, from their physiological profile (i.e., set of quantitative trait values) by comparing that profile to the trait values of other participants in the study (“training data”). For example, a 50-year-old man might have a physiological profile (values of body mass index, pulse wave velocity, NEOPIR assertiveness, etc.) that predicts a physiological age of 48 years. As a result, he would have PAR = 48/50 = 0.96 − i.e., aging slower than expected for his chronological age peers.

### Comparison of machine learning models

We applied various machine learning models to predict age from the SardiNIA trait data. Each machine learning model was cross-validated for 10,000 different training-testing splits to ensure convergent values for individuals. Physiological age was then computed as the mean predicted age for an individual across all splits for which the individual was in the testing set. The physiological aging rate (PAR) was calculated as the ratio of physiological age to chronological age. The average PAR and median PAR are close to one (SardiNIA mean PAR = 1.04, median PAR = 1.00). Slowed and accelerated aging correspond to PAR < 1 and PAR > 1 respectively.

**Figure 1:**
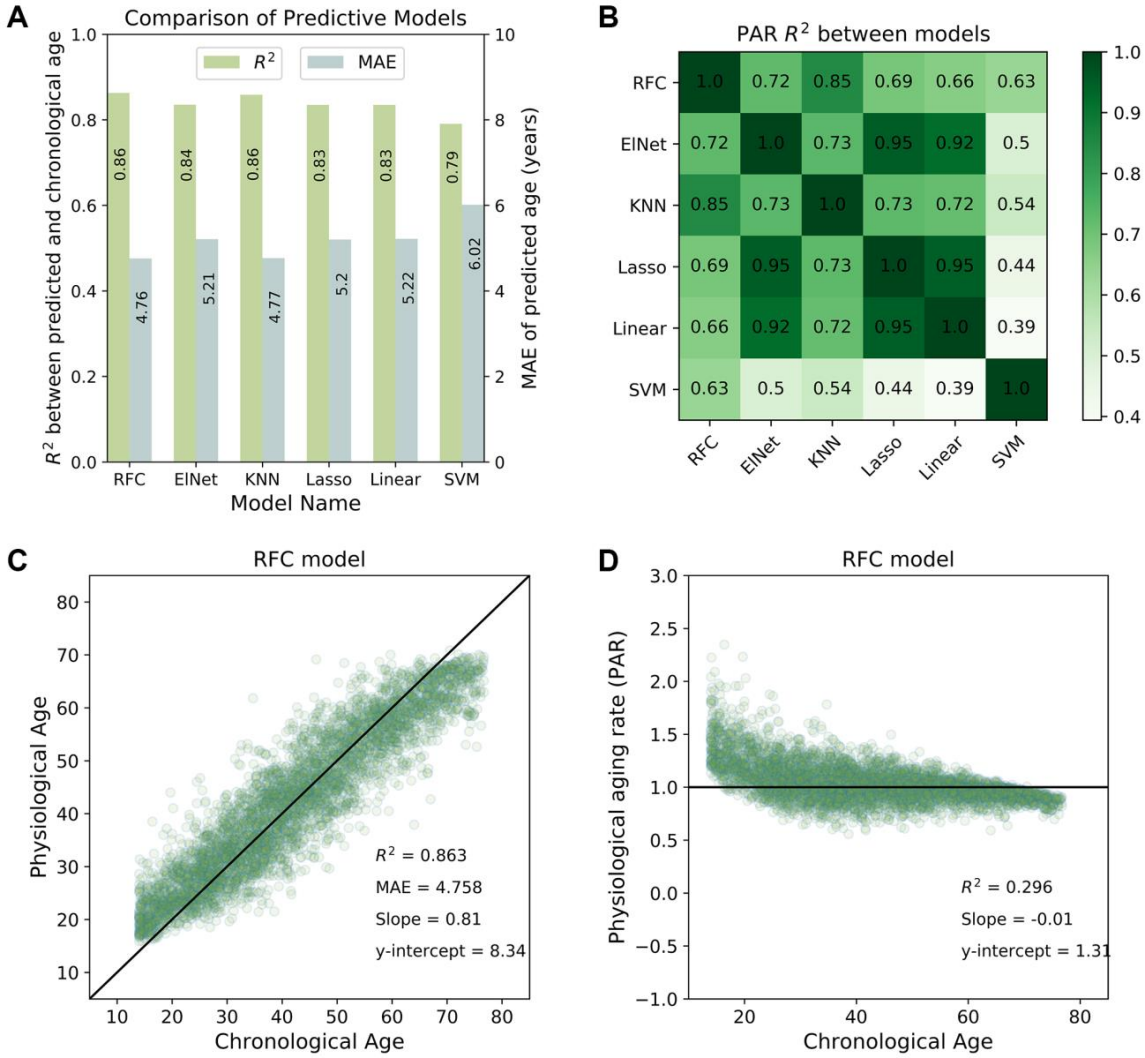
**Predictive performance of machine learning models.** (**A**) Comparison of the predictive performance measured by the coefficient of determination (R^2^) and mean absolute error (MAE) for all machine learning models investigated in the study. The random forest classifier (RFC) model was among the top-performing models. (**B**) Physiological aging rates were highly correlated between different models. Shown is a tileplot of R^2^ between PARs obtained from different models where darker green corresponds to higher values. (**C**) Physiological ages predicted by the RFC model were well-correlated with chronological ages of individuals in the SardiNIA study. (**D**) Physiological aging rate (PAR) of individuals obtained from the RFC model was weakly correlated with chronological age. All figures shown are for the baseline (W1) SardiNIA study. Similar results as in (**C**) and (**D**) were observed in follow-up waves of the SardiNIA study, for the elastic net regression model, and in the InCHIANTI study (see Supplementary Materials).

We used the coefficient of determination (R^2^) between the actual and predicted ages to evaluate the performance of each model. Higher R^2^ values indicated better model performance, with most individuals having concordant chronological and physiological age. All machine learning models produced well-correlated physiological age estimates (R^2^ > 0.8; see Figure 1A). The physiological age and PAR measurements were also highly correlated between any two machine learning models (R^2^ > 0.9 for physiological ages; R^2^ > 0.7 for PARs; see Figure 1B). These similarities suggest the presence of a model-agnostic, intrinsic physiological aging signal. The top-performing model, the random forest classifier (RFC) (R^2^ = 0.86 for the SardiNIA baseline study; see Figure 1C), reached performance saturation with 37 rank-ordered traits when all individuals were involved in training/testing, or with 75 individuals when all traits were involved in training/testing (see Supplementary Figure 3).

Results were consistent between the baseline study and all follow-up waves of the SardiNIA study and were also consistent between all study visits in the InCHIANTI study (see Supplementary Figure 4 and Supplementary Figure 5 respectively). In the baseline study, the standard deviation in predicted age was 2.6 years for SardiNIA and 3.3 years for InCHIANTI, and standard deviation in predicted age was uncorrelated with subject age for both studies. In SardiNIA, there was no significant difference between the PAR measures of male and female study participants (see Supplementary Figure 6). The distribution of PARs obtained from the RFC model was also generally independent of age (Figure 1D) and highly similar between the SardiNIA and InCHIANTI studies despite notable differences in the chronological age range of the two cohorts (see Supplementary Materials, Supplementary Figure 7). However, the very youngest individuals were distinctly more likely to have PAR > 1, and the very oldest individuals were more likely to have PAR < 1. This trend may be due to several factors: selection bias, with individuals who survived to advanced age being more likely to have a slower intrinsic aging rate; different underlying biology for the youngest individuals who are undergoing development; or artefacts in the classification procedure caused by the absence of data for individuals younger than the youngest or older than the oldest in the cohort (see “Linear rescaling and trimming to correct for the imbalanced distribution of PARs across age” in Supplementary Methods for details on linear rescaling and/or trimming of predicted ages to reduce the PAR imbalance). Removing the youngest and oldest age bins resulted in a more balanced distribution of PARs across chronological age but did not significantly change the correlation of physiological age with chronological age in either SardiNIA or InCHIANTI (see Supplementary Figure 8).

### Using common traits

Models for estimating physiological age are especially useful if they can work with commonly measured traits. Therefore, we trained the RFC model on a reduced set of SardiNIA traits that are part of routine clinical screenings. The physiological age obtained from this model was again strongly correlated with chronological age (R^2^ = 0.78 for RFC). The addition of ten cardiovascular biomarkers that are commonly measured in cardiology specialty clinics resulted in a total of 65 traits (52 after Fisher trait selection, see Methods; full list in Supplementary Materials) and further increased model performance to a level comparable to the full-trait model (R^2^ = 0.85 for random forest on the baseline SardiNIA data, see Supplementary Materials). The physiological age and PAR measurements obtained from this “common-trait model” correlated well with their counterparts from the full-trait model (R^2^ = 0.95 for physiological age; R^2^ = 0.8 for PAR, see Supplementary Figure 9). These results are consistent with a clinically relevant intrinsic process of aging (see Discussion). Results for the common-trait model were also consistent for follow-up waves of SardiNIA (see Supplementary Figure 10).

### Physiological aging rate is associated with mortality

Next, we assessed the clinical relevance of the new aging rate measurement. Specifically, we tested whether PAR is associated with mortality using two methods. First, a Cox proportional hazards model showed that independent of chronological age and sex, PAR was a significant predictor for survival (263 deaths, ~51,000 person-years). As shown in Table 1, a two-fold higher aging rate was significantly associated with a 3.59 fold increase of the death hazard (*p*-value = 0.010). As age and sex were included as covariates in the model, we also observed that independent of PAR, a one-year age increase would increase the hazard by 1.11 fold (*p*-value <2 × 10^−16^) and that males have a hazard 1.67 times higher than females (*p*-value = 6 × 10^−5^). As a point of comparison, we used the well-known Klemera-Doubal method (KDM) to compute biological age using six traits: C-reactive protein, creatinine, glycated hemoglobin, total cholesterol, urea nitrogen, and systolic blood pressure [37]. Unlike PAR, the KDM biological aging rate was not significantly associated with mortality (*p* = 0.53, Cox regression with age, sex, log_2_ (KDM aging rate); 275 deaths, ~26,800 person-years).

**Table 1:** The effect of physiological aging rate (and age and sex) on survival from a cox proportional hazards model.

	**Coefficient**	**Exp (Coefficient) -- hazard ratio**	**z-statistic**	***p*-value**
Age	0.11	1.11	15.69	<2e-16
Sex	0.51	1.67	4.02	5.9e-5
log_2_(PAR)	1.28	3.59	2.57	0.010

**Figure 2:**
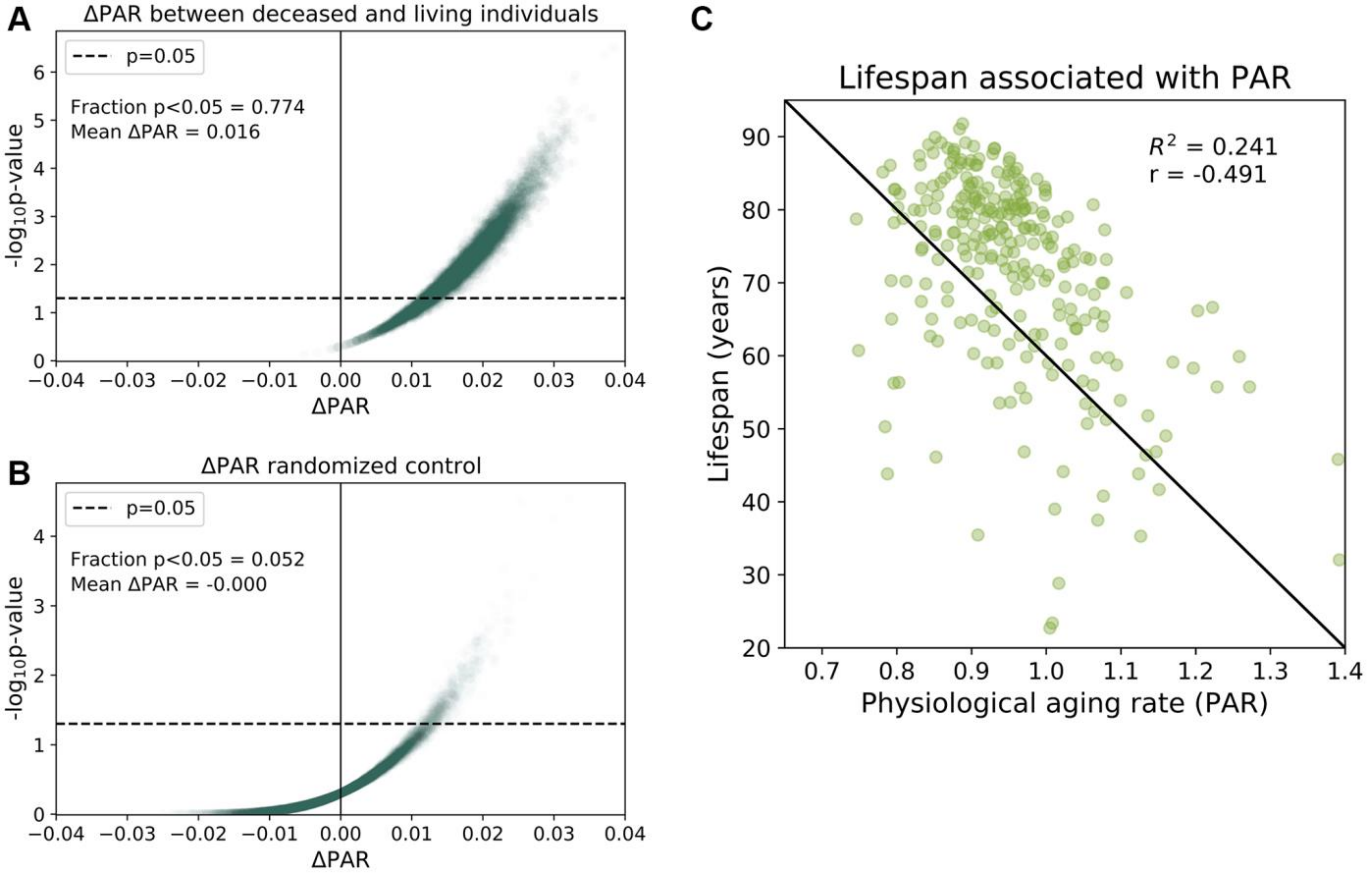
**Physiological aging rates are associated with mortality.** (**A**) Age-matched mortality analysis: 265 deceased participants were randomly paired with age- Matched ± 0.5 years) living participants in the baseline SardiNIA study. We calculated the difference in the mean PAR measurements of the two groups, ΔPAR = PAR_deceased_ – PAR_living_ and the corresponding *p*-value from a one-sided, one-sample *t*-test for ΔPAR > 0.The age-matched grouping was performed 10000 times and ΔPAR and *p*-values were calculated for each of the 10000 comparisons. 77.4% of the age-matched comparisons produced significantly greater than zero ΔPAR values (*p* < 0.05) and the mean ΔPAR across all comparisons was 0.016. Nearly all comparisons (>99%) had ΔPAR > 0, which indicated that PAR_deceased_ > PAR_living_ on average. (**B**) Randomized, age-matched control comparisons produced a 5.2% frequency of significantly greater than zero (*p* < 0.05) ΔPAR values and the mean ΔPAR was 0.00. Consistent with random assignment, 50.8% of the ΔPAR values were greater than zero. (**C**) Lifespans for individuals were negatively correlated with PARs (r = −0.491).

To provide more evidence for the association between PAR and mortality, we then randomly age-matched deceased individuals with living participants and compared their aging rates. We performed 100,000 randomizations and found that 77.4% of the age-matched comparisons had significant differences (*p* < 0.05) in the mean PAR values of the deceased and living subjects with a mean ΔPAR of 0.016 (Figure 2A). Nearly all random age-matched comparisons reported ΔPAR > 0 (Figure 2A). In contrast, the randomized control showed, as expected under null, few (5.2%) *p*-values < 0.05 and a mean ΔPAR = 0.00 (Figure 2B). Furthermore, lifespans of these individuals were negatively correlated with the corresponding PARs in both the full-trait model (r = −0.491, Figure 2C) and common-trait model (r = −0.469, see Supplementary Figure 11). These results all suggest that individuals with lower PARs outlived their counterparts with higher PARs.

### Correlation of physiological aging rate (PAR) with epigenetic aging rate (EAR)

**Figure 3:**
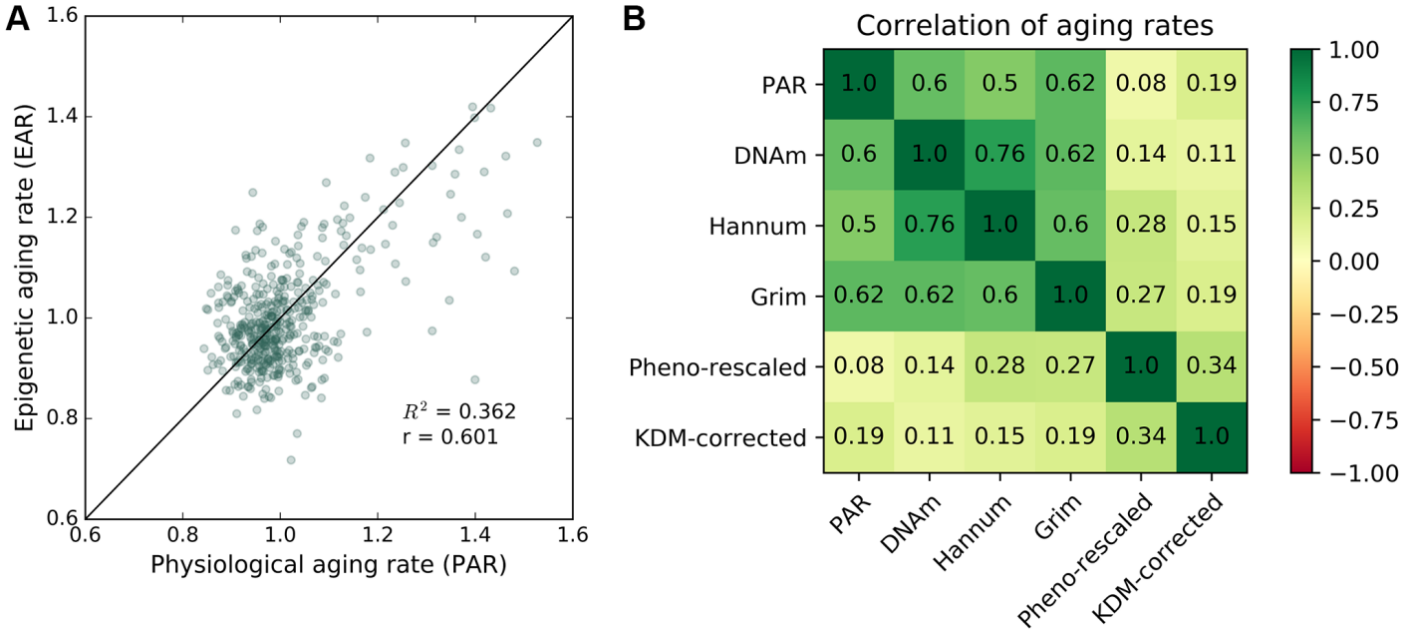
**Correlation between epigenetic aging rate and physiological aging rate.** Correlation between PAR and other aging rate measures. (**A**) Mean physiological aging rates (PARs) obtained from physiological age measurements were correlated (R^2^ = 0.362, r = 0.601) with the mean epigenetic aging rates (EARs) calculated for the same individuals across the baseline and latest follow-up InCHIANTI studies using the Horvath DNAm age. (**B**) Pearson correlation values between different pairings of aging rate measures including PAR, Horvath DNAm age, the Hannum DNAm age, GrimAge, rescaled PhenoAge, and the corrected KDM biological age with eight covariates.

DNA methylation (DNAm) ages were measured for individuals in the baseline study and the most recent follow-up study of InCHIANTI with the Horvath model [20]. Using the same method for calculating PAR, we calculated a DNA methylation-based epigenetic aging rate (EAR) as the ratio between the DNAm age and the chronological age for an individual. The mean EAR was correlated with the mean PAR (R^2^ = 0.36, r = 0.6, Figure 3A) despite the two modalities representing measures of aging from separate biological levels (i.e., methylome for EAR; proteome, metabolome, and phenome for PAR). The positive correlation between EAR and PAR persisted after eliminating the effect of chronological age and sex as confounding variables (see Supplementary Figure 12). Both PAR and EAR measurements were largely uncorrelated with chronological age. In addition to DNA methylation-based aging rates from [20], the PAR was also positively correlated with DNA methylation-based aging rates [19], GrimAge aging rates [24], and PhenoAge aging rates [23] (see Figure 3B). We also computed the KDM biological age [37] using eight markers from InCHIANTI, which were C-reactive protein, creatinine, glycated hemoglobin, albumin, total cholesterol, alkaline phosphatase, urea nitrogen, and systolic blood pressure. The PAR was better correlated than KDM biological aging rate with all DNA methylation-based aging rates except PhenoAge, which suggests that the PAR is a relatively good proxy for DNA methylation-based aging measures.

### Heritability and GWAS of physiological aging rate

We estimated the extent to which PAR is heritable using pedigrees. PARs from the full-trait RFC model observed moderate heritability (h^2^ = 0.33), and PARs obtained using the common-trait model were similarly heritable (h^2^ = 0.30). These values were comparable to previous estimates for the heritability of aging and longevity (Karasik et al., 2005). Furthermore, modifications of the PAR measurements such as linear rescaling of physiological ages increased heritability up to h^2^~0.45 (see Supplementary Materials and Supplementary Figure 13; see Supplementary Figure 14 for linearly rescaled physiological age results). Like typical traits, the physiological aging rate, a proxy for the intrinsic aging process, appears to have a significant genetic component.

**Figure 4:**
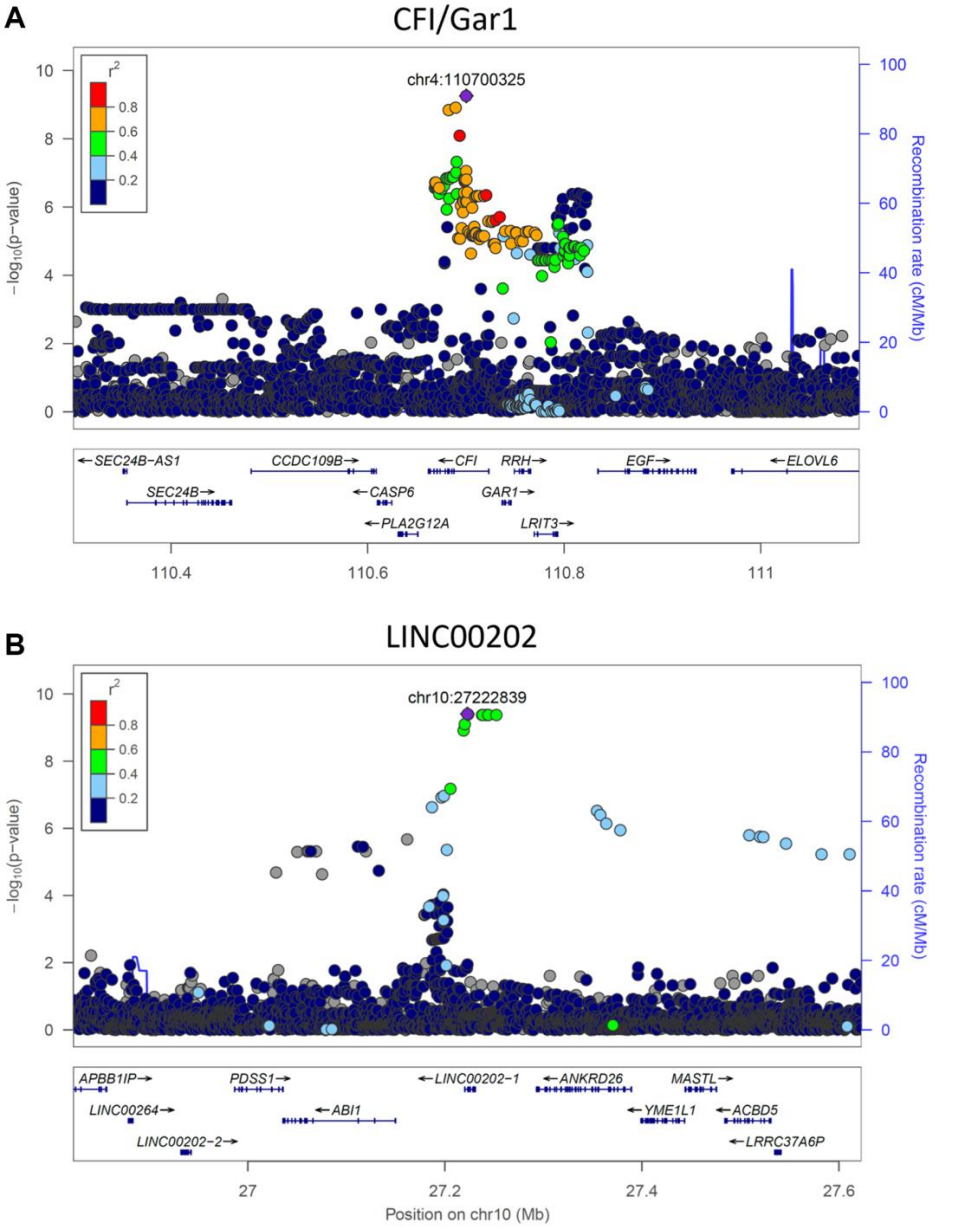
**Significant genetic loci obtained from genome-wide association study.** (**A**) *CFI*/*GAR1* was significantly associated with the physiological aging rate (PAR). CFI is a complement factor and has been linked to age-related macular degeneration and other age-related disorders. GAR1 is an accessory protein for the active telomerase complex and is an eQTL target of the top *CFI*/*GAR1* SNPs. (**B**) *LINC00202* was significantly associated with the PAR and corresponded to a long non-coding RNA that has been indirectly linked to age-related disease. Plots were made using LocusZoom (Pruim et al., 2010).

The heritability of PAR encouraged us to perform genome-wide association studies (GWAS) in SardiNIA using PAR as a quantitative trait. GWAS identified two genome-wide significant loci [16 significant (*p* < 5.00E-8) single-nucleotide polymorphisms] associated with PAR. Six SNPs fell in the *CFI*/*GAR1* locus located on chromosome 4 (Figure 4A, most significant *CFI*/*GAR1* SNP with *p* = 5.55E-10), which has already been linked to aging in earlier studies (see Discussion). *CFI*/*GAR1* also harbored the most significant SNP associated with physiological age acceleration (PAA), another measure of age progression (*p* = 4.07E-9) (see Supplementary Materials), and was among the top SNPs associated with the PAR from the common-trait model (*p* = 1.731E-7) and with other machine learning models (e.g., elastic net regression model, *p* = 2.95E-7, see Supplementary Materials).

Three significant SNPs and seven significant proximally located SNPs (*p* < 5.00E-8) were observed at the second locus, *LINC00202*, also referred to as *FAM238C* (Figure 4B, most significant *LINC00202* SNP with *p* = 3.94E-10). The *LINC00202* locus also contained the top SNPs for PAR measurements from the common-trait model (*p* = 1.100E-8) and from other machine learning models (e.g., elastic net regression, *p* = 2.153E-10). *LINC00202* is a long intergenic non-coding RNA with largely uncharacterized function (see Discussion). Other genes with suggestive *p*-values for possible associations with PAR (*p* < 5.00E-7) include *APLF*, *ARHGAP15*, *SCD5*, *SOGA2*, *HIVEP1*, *ANKRD26*, *DYNC2LI1*, *ZNF518B*, *TMEM45A*, *CLDN10*, and *CSMD1* (see Supplementary Materials for a discussion of these genes).

### Trait ranking and biomarkers of aging

**Figure 5:**
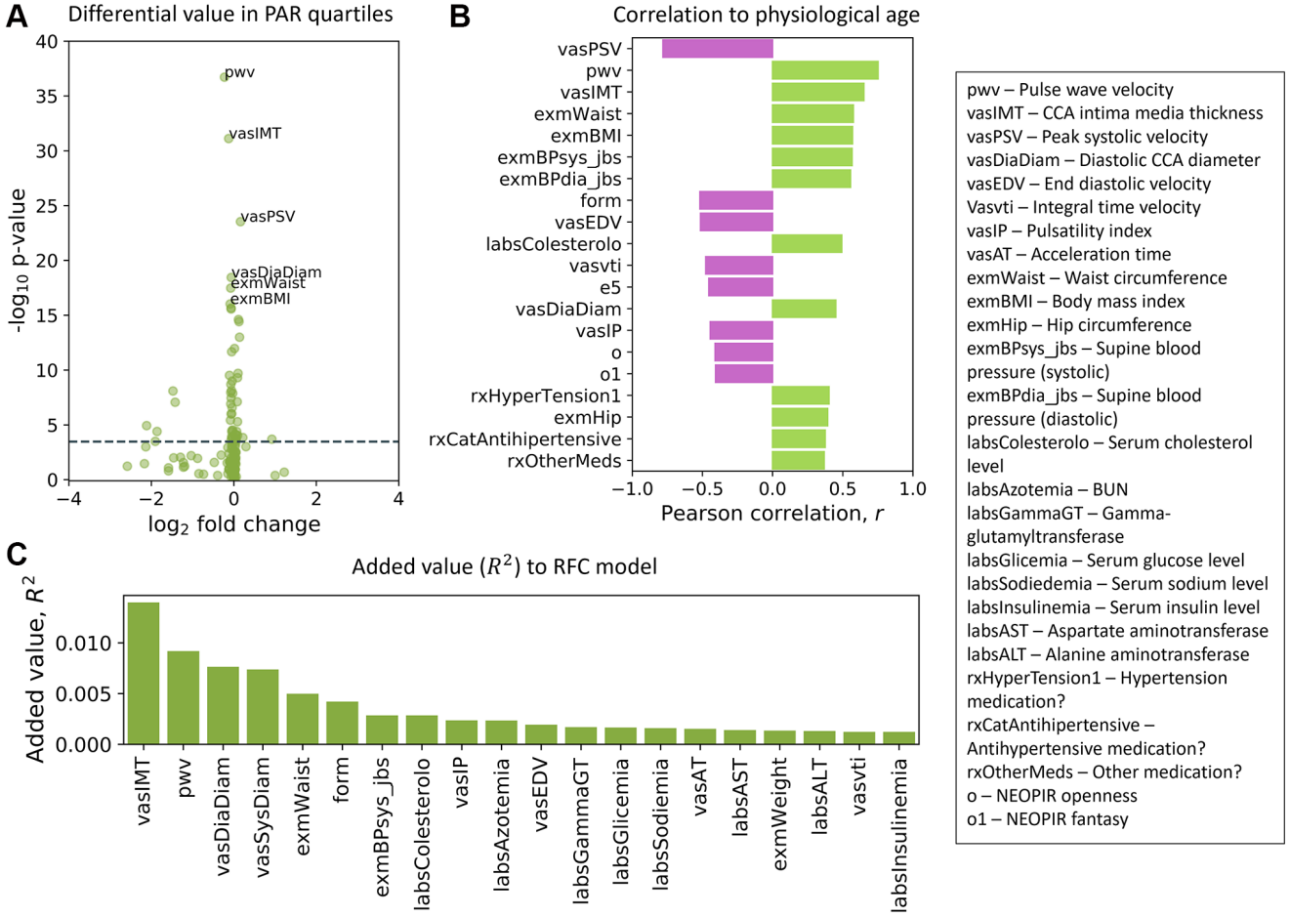
**Top traits determined by three independent methods.** (**A**) Volcano plot of the top traits in the full-trait model, which included pulse wave velocity (pwv), CCA intima media thickness (vasIMT), peak systolic velocity (vasPSV), diastolic CCA diameter (vasDiaDiam), waist circumference (exmWaist), and body mass index (exmBMI). Significant differences between the mean trait values of subjects in the top and bottom PAR quartiles were determined using a two-tailed students *t*-test on each trait. The dotted line corresponds to a Bonferroni-corrected threshold of *p* = 3.33 × 10^−4^ calculated from single-test threshold of *p* = 5.00 × 10^−2^. Many top traits were also highly ranked in the common-trait model. (**B**) Traits rank-ordered by Pearson correlation (r) with physiological age measured using the full-trait RFC model. (**C**) Traits rank-ordered by approximate added value (R^2^) for the full-trait RFC model in SardiNIA (R^2^ = 0.858 with all traits used in model). Added value (R^2^) was averaged over 500 training-testing iterates for each trait. There was significant overlap in the highest ranked traits across all three scoring methods and between the common-trait and full-trait models (see Supplementary Materials). Trait names are as they appear in the SardiNIA study; descriptions of all traits are available in the Supplementary Materials.

We ranked the traits in the SardiNIA study and separately for the InCHIANTI study using three distinct methods (see Methods). These included measuring the difference in trait values between the top and bottom PAR quartiles (Figure 5A), correlation of traits with physiological age (Figure 5B), and the additional contribution of each trait to model performance (Figure 5C), which is similar to variable importance for random forest models [63]. Although, as outlined in the Methods, the sets of traits used in SardiNIA and InCHIANTI were not identical, the top ranked traits showed general concordance in the two studies. For example, a set of cardiovascular traits were ranked as top traits in both cohorts. In SardiNIA, CCA intima media thickness, pulse wave velocity, and diastolic CCA diameter were highly ranked across all three methods (Figure 5). Several other cardiovascular traits also received high scores, including peak systolic velocity, integral time velocity, end diastolic velocity, and mean supine systolic blood pressure. Most of these traits were also highly ranked among the set of common and cardiovascular SardiNIA traits (Spearman correlation of *ρ* = 0.92, see Supplementary Figure 15), corroborating the comparable predictive performance between the common-trait and the full-trait models. The prevalence of high-scoring cardiovascular traits was likewise seen in the InCHIANTI study cohort, including pulse wave velocity, systolic blood pressure, and repolarization phase among the top 5% of ranked traits.

Some NEOPIR (Revised NEO Personality Inventory) personality traits [64] were highly ranked in SardiNIA, including fantasy, compliance, excitement seeking, and assistance with personality test. Other highly ranked SardiNIA traits across multiple methods included general measures of obesity such as waist circumference and body mass index, and blood molecular markers such as cholesterol, nitrogen, glucose, sodium, uric acid, and alanine aminotransferase levels. Many of these traits were also present among the highest-ranking InCHIANTI traits (see Supplementary Figure 16: in the top 5%: blood nitrogen, creatinine, sodium intake, hypertension; in the top 20%: cholesterol, IL-6, fibrinogen; and in the top 30%: uric acid, transferrin, waist circumference). In particular, creatinine level was highly ranked by added value (R2) to model performance and was also the highest-ranked trait in the InCHIANTI study. InCHIANTI trait rankings (see Methods) using the epigenetic age and EAR resulted in similar sets of high-ranked features (see Supplementary Figure 17). Most of the top traits from each scoring method have been suggested earlier as biomarkers of aging, and others may become candidate biomarkers (see Supplementary Materials for full rankings of traits).

## DISCUSSION

We present machine learning as a promising framework for measuring physiological age from broad-ranging physiological, cognitive, and molecular traits.

### Physiological aging rate model

The measured physiological age is well-correlated with chronological age (R^2^ > 0.8), and different sets of traits give very comparable estimates of physiological aging rate (PAR). Furthermore, PAR calculated from the traits is correlated with the epigenetic aging rate (EAR) (R^2^ = 0.36, r = 0.6), even though the two aging rates are based on separate biological frameworks. Comparisons with aging rates derived from newer epigenetic age models, such as GrimAge [24] and phenotypic epigenetic age [22], could provide further insight into this relationship.

The physiological age estimates obtained from the model are well-correlated with chronological age and comparable to epigenetic age measures. The approach described here joins other recent attempts to quantify “aging” from functional parameters. It is encouraging that our results correlate with other approaches that are also predictive of risk of mortality. In particular, the PAR, as compared to the KDM biological aging rate, is better correlated with GrimAge, the epigenetic age score with the strongest mortality association [65]. Unlike many earlier studies on aging metrics, we show that PAR is longitudinally stable for an individual (see Supplementary Figure 18), with temporal correlation comparable to that of epigenetic age acceleration indices [66] and higher for shorter time differences, which is a necessary and desirable property for biological age measurements [3, 5, 6]. Also, because PAR predictions are derived from multiple physiological, cognitive, and molecular inputs that span the proteome, metabolome, and phenome, they potentially represent a larger fraction of the manifestations of aging than earlier models. The model maintains high predictive performance even after reducing the trait set to 65 commonly accessible markers. These traits can be efficiently and inexpensively measured in many clinics already equipped to collect them—making the model framework, like some other current models [37], easily adaptable for clinical studies and workflows.

We focused primarily on the random forest classifier model in this investigation, but all machine learning models detected a strong physiological aging signal (R^2^ > 0.80). The strength of the signal corroborates the presence of a central aging effect that exists independently of model type. This is an idea that has been previously explored in theoretical models [67, 68] and studies where temporal scaling of a central aging rate was observed [69]. The top traits we found in both SardiNIA and InCHIANTI studies are manifest in many tissues and organs. The findings are thus consistent with the likelihood that PAR represents the rate of a pathology-independent aging process—an answer to Medawar’s famous question [1].

Top traits identified through various methods recapitulate previously identified associations between physiological aging and/or age-related disease and cardiovascular function [70–72], molecular markers, physiological measurements, and personality features. A few novel exceptions are promising candidates for new biomarkers of aging (e.g., NEOPIR compliance and agreeableness, step-climbing endurance, and hand tapping coordination).

### PAR as a predictor of survival

Improved prediction of chronological age is not necessarily associated with increased prediction of mortality [73]. We used two methods to test directly for association between PAR and mortality/survival. As shown by a Cox proportional hazards model, a two-fold increase of the aging rate would increase the risk of dying by 3.59 fold, or equivalently, a 10% increase of aging rate would increase the risk by 1.19 fold. Our second method showed that in 100,000 randomizations, deceased individuals had significantly higher PARs (*p* < 0.05) than age-matched living subjects 77.4% of the time. These results clearly show the effect of aging rate on survival and thus a prospective benefit of slowing aging rates. In the same Cox survival model, we also observed the effects of age and sex on survival (e.g., a one-year age increase would increase the risk of dying by 1.11 fold on average, and males have a risk 1.67 times higher than females of the same-age). These results are consistent with numbers from other resources. For example, based on the 2017 Actuarial Life Table published by US Social Security Administration, a 50-year-old man has a death probability 1.09 times higher than a 49-year-old man, and a 50-year-old man has a death probability 1.60 times higher than a 50-year-old woman. These consistent results support the validity of the apparent effect of PAR on survival. In comparison, aging rates computed using the Klemera-Doubal method, another non-epigenetic biological age model, were not significantly associated with mortality.

### Heritability and GWAS signals for PAR

The heritability of PAR (~30%) implies a significant genetic contribution to an individual’s aging rate. Genome-wide association studies identified the *CFI*/*GAR1* and *LINC00202* loci to be significantly associated with the physiological aging rate. Both loci have also been previously associated with aging and age-related disease.

Complement factor I (CFI) is involved in eliminating pathogens, triggering inflammation, and removing debris from cells and tissues [74–76]. Variants in the CFI gene have been linked to age-related macular degeneration (AMD) [75, 77–80], chronic infection [81], and broader aging processes [77, 82].

But intriguingly, GAR1, encoded by the other candidate gene at the locus, is one of four accessory proteins associated with the telomerase ribonucleoprotein (RNP) complex [83]. *GAR1* polymorphisms have been associated with differences in acute heart rate response to exercise, which is a predictor of all-cause mortality and cardiovascular mortality [84]. Other components and effectors of telomerase have also been linked to aging [60, 62, 85, 86].

We retrieved information on significant single-tissue expression quantitative trait loci (eQTLs) for *CFI* and *GAR1* mRNAs from the GTEx database [87]. All significant *CFI*/*GAR1* SNPs corresponding to significant eQTLs were associated with the expression level of *GAR1* mRNA, but not with that of *CFI*. Further, *GAR1* mRNA was the most significantly associated (*p* = 6.5E-13) for the eQTL corresponding to the top *CFI*/*GAR1* SNP (*rs11940869*) across all tissues. Significant eQTLs corresponding to *rs11940869* were found in several tissue types, including brain (frontal cortex, caudate, putamen) and esophagus mucosa (see Supplementary Materials). Furthermore, we analyzed RNA-seq gene expression profiles of tissues from six diverse GTEx tissues (heart, lung, liver, thyroid, colon, cerebellum). *GAR1* mRNA levels were reduced with age in heart (r = −0.135), lung (r = −0.313), and colon (r = −0.231) tissues, whereas *CFI* mRNA expression levels did not correlate substantially with age in most human organs (see Supplementary Figure 19). These results suggest that it may not be coincidental that telomerase, which is associated with GAR1, was determined to be the critical factor that prevents replicative senescence in the fibroblast model for aging: the supply of active telomerase to aging cells promotes their indefinite growth and division, without activation of aging-related SASP (senescence-associated secretory phenotype) proteins. Our data suggest that effects on telomerase activity and possibly related effects on chromatin are not only markers for aging but may be part of the intrinsic mechanism, implicating cell senescence.

As for the other genome-wide significant signal in *LINC00202*, expression quantitative trait loci affecting *LINC00202* expression have been significantly associated with age-related endophenotypes in earlier genome-wide association studies [88]. Although the role of most lncRNAs remains unknown, a number of lncRNAs have been shown to influence molecular processes that contribute to age-related phenotypes [89], and are known to influence age-related cardiovascular disease [90], oxidative stress responses and cellular senescence [91], and many age-related debilitations [92]. However, an exact role of *LINC00202* in the aging process remains to be determined.

### Possible next steps

We used data from two independent longitudinal studies (SardiNIA and InCHIANTI) with relatively homogenous populations. Whether the significantly PAR-associated loci and highest-scoring traits reported in this investigation are reproducible in more heterogenous populations must be investigated. Further hyperparameter tuning and recent developments in machine learning for handling sparse data [93] may also be of value in maximizing the utility of trait data for age prediction. Although PAR is measured from molecular, physiological, and cognitive traits, inclusion of traits from other data modalities may improve the metric. Several modalities that are aging-associated and potentially useful for PAR measurement include human transcriptome profiles [45, 94, 95], proteome profiles [46, 47], and image data [42, 43]. One natural extension of our method, which is based on cross-sectional trait data, is to use longitudinal data to predict a physiological age at a subsequent visit (e.g., to predict the aging rate at a subsequent visit based on the change of traits over time or based directly on the aging rates predicted at previous visits). This involves new method development and deserves efforts from a separate study.

Although we have emphasized the genetic contribution to aging rates, the majority of PAR is still attributable to environmental factors, including the effect of pathology. Further investigation of correlations between PAR and smoking or age-related diseases such as diabetes, neurodegenerative disease, cancer, and cardiovascular disease could further aid in characterizing the highest-scoring traits and delineating potential aging-related pathways. In turn, these analyses may offer additional clinical utility for PAR in assessing the personalized risk and natural history of patients for each disease.

The efficacy of treatments aimed at slowing the aging process has traditionally been evaluated using individual biomarkers or limited collections of related biomarkers. Our current study has shown that PAR is a significant predictor for survival and correlated with epigenetic aging rate, providing evidence for a good measurement of “aging”. Therefore, we see two potential clinical applications of this intuitive metric—the physiological aging rate: 1) physicians might evaluate the efficacy of treatments and interventions on the aging process by the change of PAR before and after the treatments; 2) based on the values of traits that contribute to the aging rate, physicians can make recommendations to individuals aimed at slowing the aging process (e.g., making specific diet changes, doing specific exercises). Notably, the recommendations would be much the same that are currently recommended by physicians, but the additional link of PAR to risk of mortality might motivate patients to overcome the typical reluctance to modify their regimen.

## Supplementary Materials

Supplementary Methods

Supplementary Figures

Supplementary Table 1

## References

[1] Medawar PB. An Unsolved Problem in Biology. HK Lewis Co. 1952.

[2] Baker GT 3rd, Sprott RL. Biomarkers of aging. Exp Gerontol. 1988; 23:223–39. 10.1016/0531-5565(88)90025-3 3058488

[3] Ferrucci L, Gonzalez-Freire M, Fabbri E, Simonsick E, Tanaka T, Moore Z, Salimi S, Sierra F, de Cabo R. Measuring biological aging in humans: A quest. Aging Cell. 2020; 19:e13080. 10.1111/acel.13080 31833194 PMC6996955

[4] Sprott RL. Biomarkers of aging and disease: introduction and definitions. Exp Gerontol. 2010; 45:2–4. 10.1016/j.exger.2009.07.008 19651201

[5] Khan SS, Singer BD, Vaughan DE. Molecular and physiological manifestations and measurement of aging in humans. Aging Cell. 2017; 16:624–33. 10.1111/acel.12601 28544158 PMC5506433

[6] Kuo PL, Schrack JA, Shardell MD, Levine M, Moore AZ, An Y, Elango P, Karikkineth A, Tanaka T, de Cabo R, Zukley LM, AlGhatrif M, Chia CW, et al. A roadmap to build a phenotypic metric of ageing: insights from the Baltimore Longitudinal Study of Aging. J Intern Med. 2020; 287:373–94. 10.1111/joim.13024 32107805 PMC7670826

[7] Ferrucci L, Levine ME, Kuo PL, Simonsick EM. Time and the Metrics of Aging. Circ Res. 2018; 123:740–44. 10.1161/CIRCRESAHA.118.312816 30355074 PMC6205734

[8] Jylhävä J, Pedersen NL, Hägg S. Biological Age Predictors. EBioMedicine. 2017; 21:29–36. 10.1016/j.ebiom.2017.03.046 28396265 PMC5514388

[9] Shamir L. Composite Aging Markers Can Be Used for Quantitative Profiling of Aging. Gerontology. 2015; 62:66–68. 10.1159/000433466 26088420

[10] Behrens YL, Thomay K, Hagedorn M, Ebersold J, Henrich L, Nustede R, Schlegelberger B, Göhring G. Comparison of different methods for telomere length measurement in whole blood and blood cell subsets: Recommendations for telomere length measurement in hematological diseases. Genes Chromosomes Cancer. 2017; 56:700–08. 10.1002/gcc.22475 28593741

[11] Benetos A, Okuda K, Lajemi M, Kimura M, Thomas F, Skurnick J, Labat C, Bean K, Aviv A. Telomere length as an indicator of biological aging: the gender effect and relation with pulse pressure and pulse wave velocity. Hypertension. 2001; 37:381–85. 10.1161/01.hyp.37.2.381 11230304

[12] Bramwell JC, Hill AV. The velocity of pulse wave in man. Proc R Soc Lond B. 1922; 93:298–306. 10.1098/rspb.1922.0022

[13] Hochstrasser T, Marksteiner J, Humpel C. Telomere length is age-dependent and reduced in monocytes of Alzheimer patients. Exp Gerontol. 2012; 47:160–63. 10.1016/j.exger.2011.11.012 22178633 PMC3278593

[14] Jiang H, Schiffer E, Song Z, Wang J, Zürbig P, Thedieck K, Moes S, Bantel H, Saal N, Jantos J, Brecht M, Jenö P, Hall MN, et al. Proteins induced by telomere dysfunction and DNA damage represent biomarkers of human aging and disease. Proc Natl Acad Sci U S A. 2008; 105:11299–304. 10.1073/pnas.0801457105 18695223 PMC2516278

[15] Rantanen T, Masaki K, Foley D, Izmirlian G, White L, Guralnik JM. Grip strength changes over 27 yr in Japanese-American men. J Appl Physiol (1985). 1998; 85:2047–53. 10.1152/jappl.1998.85.6.2047 9843525

[16] Studenski S, Perera S, Patel K, Rosano C, Faulkner K, Inzitari M, Brach J, Chandler J, Cawthon P, Connor EB, Nevitt M, Visser M, Kritchevsky S, et al. Gait speed and survival in older adults. JAMA. 2011; 305:50–58. 10.1001/jama.2010.1923 21205966 PMC3080184

[17] Wilkinson IB, Cockcroft JR, Webb DJ. Pulse wave analysis and arterial stiffness. J Cardiovasc Pharmacol. 1998 (Suppl 3); 32:S33–37. 9883745

[18] Fries JF. Aging, natural death, and the compression of morbidity. 1980. Bull World Health Organ. 2002; 80:245–50. 11984612 PMC2567746

[19] Hannum G, Guinney J, Zhao L, Zhang L, Hughes G, Sadda S, Klotzle B, Bibikova M, Fan JB, Gao Y, Deconde R, Chen M, Rajapakse I, et al. Genome-wide methylation profiles reveal quantitative views of human aging rates. Mol Cell. 2013; 49:359–67. 10.1016/j.molcel.2012.10.016 23177740 PMC3780611

[20] Horvath S. DNA methylation age of human tissues and cell types. Genome Biol. 2013; 14:R115. 10.1186/gb-2013-14-10-r115 24138928 PMC4015143

[21] Chen BH, Marioni RE, Colicino E, Peters MJ, Ward-Caviness CK, Tsai PC, Roetker NS, Just AC, Demerath EW, Guan W, Bressler J, Fornage M, Studenski S, et al. DNA methylation-based measures of biological age: meta-analysis predicting time to death. Aging (Albany NY). 2016; 8:1844–65. 10.18632/aging.101020 27690265 PMC5076441

[22] Levine ME, Lu AT, Quach A, Chen BH, Assimes TL, Bandinelli S, Hou L, Baccarelli AA, Stewart JD, Li Y, Whitsel EA, Wilson JG, Reiner AP, et al. An epigenetic biomarker of aging for lifespan and healthspan. Aging (Albany NY). 2018; 10:573–91. 10.18632/aging.101414 29676998 PMC5940111

[23] Liu Z, Kuo PL, Horvath S, Crimmins E, Ferrucci L, Levine M. A new aging measure captures morbidity and mortality risk across diverse subpopulations from NHANES IV: A cohort study. PLoS Med. 2018; 15:e1002718. 10.1371/journal.pmed.1002718 30596641 PMC6312200

[24] Lu AT, Quach A, Wilson JG, Reiner AP, Aviv A, Raj K, Hou L, Baccarelli AA, Li Y, Stewart JD, Whitsel EA, Assimes TL, Ferrucci L, Horvath S. DNA methylation GrimAge strongly predicts lifespan and healthspan. Aging (Albany NY). 2019; 11:303–27. 10.18632/aging.101684 30669119 PMC6366976

[25] Marioni RE, Shah S, McRae AF, Chen BH, Colicino E, Harris SE, Gibson J, Henders AK, Redmond P, Cox SR, Pattie A, Corley J, Murphy L, et al. DNA methylation age of blood predicts all-cause mortality in later life. Genome Biol. 2015; 16:25. 10.1186/s13059-015-0584-6 25633388 PMC4350614

[26] Petkovich DA, Podolskiy DI, Lobanov AV, Lee SG, Miller RA, Gladyshev VN. Using DNA Methylation Profiling to Evaluate Biological Age and Longevity Interventions. Cell Metab. 2017; 25:954–60.e6. 10.1016/j.cmet.2017.03.016 28380383 PMC5578459

[27] Zheng Y, Joyce BT, Colicino E, Liu L, Zhang W, Dai Q, Shrubsole MJ, Kibbe WA, Gao T, Zhang Z, Jafari N, Vokonas P, Schwartz J, et al. Blood Epigenetic Age may Predict Cancer Incidence and Mortality. EBioMedicine. 2016; 5:68–73. 10.1016/j.ebiom.2016.02.008 27077113 PMC4816845

[28] Declerck K, Vanden Berghe W. Back to the future: Epigenetic clock plasticity towards healthy aging. Mech Ageing Dev. 2018; 174:18–29. 10.1016/j.mad.2018.01.002 29337038

[29] Chen HH. Impact of Natural Sequence Variation on Aging in the Recombinant Inbred Lines of Caenorhabditis Elegans. ProQuest. 2008.

[30] López-Otín C, Blasco MA, Partridge L, Serrano M, Kroemer G. The hallmarks of aging. Cell. 2013; 153:1194–217. 10.1016/j.cell.2013.05.039 23746838 PMC3836174

[31] Wheeler HE, Kim SK. Genetics and genomics of human ageing. Philos Trans R Soc Lond B Biol Sci. 2011; 366:43–50. 10.1098/rstb.2010.0259 21115529 PMC3001305

[32] Belsky DW, Caspi A, Houts R, Cohen HJ, Corcoran DL, Danese A, Harrington H, Israel S, Levine ME, Schaefer JD, Sugden K, Williams B, Yashin AI, et al. Quantification of biological aging in young adults. Proc Natl Acad Sci U S A. 2015; 112:E4104–10. 10.1073/pnas.1506264112 26150497 PMC4522793

[33] Blodgett JM, Theou O, Howlett SE, Rockwood K. A frailty index from common clinical and laboratory tests predicts increased risk of death across the life course. Geroscience. 2017; 39:447–55. 10.1007/s11357-017-9993-7 28866737 PMC5636769

[34] Cho IH, Park KS, Lim CJ. An empirical comparative study on biological age estimation algorithms with an application of Work Ability Index (WAI). Mech Ageing Dev. 2010; 131:69–78. 10.1016/j.mad.2009.12.001 20005245

[35] Cruz-Jentoft AJ, Daragjati J, Fratiglioni L, Maggi S, Mangoni AA, Mattace-Raso F, Paccalin M, Polidori MC, Topinkova E, Ferrucci L, Pilotto A, and MPI_AGE Investigators. Using the Multidimensional Prognostic Index (MPI) to improve cost-effectiveness of interventions in multimorbid frail older persons: results and final recommendations from the MPI_AGE European Project. Aging Clin Exp Res. 2020; 32:861–68. 10.1007/s40520-020-01516-0 32180170 PMC12159427

[36] Klemera P, Doubal S. A new approach to the concept and computation of biological age. Mech Ageing Dev. 2006; 127:240–48. 10.1016/j.mad.2005.10.004 16318865

[37] Levine ME. Modeling the rate of senescence: can estimated biological age predict mortality more accurately than chronological age? J Gerontol A Biol Sci Med Sci. 2013; 68:667–74. 10.1093/gerona/gls233 23213031 PMC3660119

[38] Mitnitski A, Collerton J, Martin-Ruiz C, Jagger C, von Zglinicki T, Rockwood K, Kirkwood TB. Age-related frailty and its association with biological markers of ageing. BMC Med. 2015; 13:161. 10.1186/s12916-015-0400-x 26166298 PMC4499935

[39] Pilotto A, Custodero C, Maggi S, Polidori MC, Veronese N, Ferrucci L. A multidimensional approach to frailty in older people. Ageing Res Rev. 2020; 60:101047. 10.1016/j.arr.2020.101047 32171786 PMC7461697

[40] Baldi P, Brunak S. Bioinformatics: The Machine Learning Approach. 2nd ed. Cambridge, MA, USA: MIT Press; 2001.

[41] Putin E, Mamoshina P, Aliper A, Korzinkin M, Moskalev A, Kolosov A, Ostrovskiy A, Cantor C, Vijg J, Zhavoronkov A. Deep biomarkers of human aging: Application of deep neural networks to biomarker development. Aging (Albany NY). 2016; 8:1021–33. 10.18632/aging.100968 27191382 PMC4931851

[42] Bobrov E, Georgievskaya A, Kiselev K, Sevastopolsky A, Zhavoronkov A, Gurov S, Rudakov K, Del Pilar Bonilla Tobar M, Jaspers S, Clemann S. PhotoAgeClock: deep learning algorithms for development of non-invasive visual biomarkers of aging. Aging (Albany NY). 2018; 10:3249–59. 10.18632/aging.101629 30414596 PMC6286834

[43] Cole JH, Ritchie SJ, Bastin ME, Valdés Hernández MC, Muñoz Maniega S, Royle N, Corley J, Pattie A, Harris SE, Zhang Q, Wray NR, Redmond P, Marioni RE, et al. Brain age predicts mortality. Mol Psychiatry. 2018; 23:1385–92. 10.1038/mp.2017.62 28439103 PMC5984097

[44] Shamir L, Wolkow CA, Goldberg IG. Quantitative measurement of aging using image texture entropy. Bioinformatics. 2009; 25:3060–63. 10.1093/bioinformatics/btp571 19808878 PMC3167693

[45] Fleischer JG, Schulte R, Tsai HH, Tyagi S, Ibarra A, Shokhirev MN, Huang L, Hetzer MW, Navlakha S. Predicting age from the transcriptome of human dermal fibroblasts. Genome Biol. 2018; 19:221. 10.1186/s13059-018-1599-6 30567591 PMC6300908

[46] Johnson AA, Shokhirev MN, Wyss-Coray T, Lehallier B. Systematic review and analysis of human proteomics aging studies unveils a novel proteomic aging clock and identifies key processes that change with age. Ageing Res Rev. 2020; 60:101070. 10.1016/j.arr.2020.101070 32311500

[47] Tanaka T, Biancotto A, Moaddel R, Moore AZ, Gonzalez-Freire M, Aon MA, Candia J, Zhang P, Cheung F, Fantoni G, Semba RD, Ferrucci L, and CHI consortium. Plasma proteomic signature of age in healthy humans. Aging Cell. 2018; 17:e12799. 10.1111/acel.12799 29992704 PMC6156492

[48] Pilia G, Chen WM, Scuteri A, Orrú M, Albai G, Dei M, Lai S, Usala G, Lai M, Loi P, Mameli C, Vacca L, Deiana M, et al. Heritability of cardiovascular and personality traits in 6,148 Sardinians. PLoS Genet. 2006; 2:e132. 10.1371/journal.pgen.0020132 16934002 PMC1557782

[49] Scuteri A, Najjar SS, Orrú M, Albai G, Strait J, Tarasov KV, Piras MG, Cao A, Schlessinger D, Uda M, Lakatta EG. Age- and gender-specific awareness, treatment, and control of cardiovascular risk factors and subclinical vascular lesions in a founder population: the SardiNIA Study. Nutr Metab Cardiovasc Dis. 2009; 19:532–41. 10.1016/j.numecd.2008.11.004 19321325 PMC4658660

[50] Cesari M, Penninx BW, Pahor M, Lauretani F, Corsi AM, Rhys Williams G, Guralnik JM, Ferrucci L. Inflammatory markers and physical performance in older persons: the InCHIANTI study. J Gerontol A Biol Sci Med Sci. 2004; 59:242–48. 10.1093/gerona/59.3.m242 15031308

[51] Ferrucci L, Bandinelli S, Benvenuti E, Di Iorio A, Macchi C, Harris TB, Guralnik JM. Subsystems contributing to the decline in ability to walk: bridging the gap between epidemiology and geriatric practice in the InCHIANTI study. J Am Geriatr Soc. 2000; 48:1618–25. 10.1111/j.1532-5415.2000.tb03873.x 11129752

[52] Cousminer DL, Berry DJ, Timpson NJ, Ang W, Thiering E, Byrne EM, Taal HR, Huikari V, Bradfield JP, Kerkhof M, Groen-Blokhuis MM, Kreiner-Møller E, Marinelli M, et al, and ReproGen Consortium, and Early Growth Genetics (EGG) Consortium. Genome-wide association and longitudinal analyses reveal genetic loci linking pubertal height growth, pubertal timing and childhood adiposity. Hum Mol Genet. 2013; 22:2735–47. 10.1093/hmg/ddt104 23449627 PMC3674797

[53] Ho TK. Random Decision Forests. Proceedings of 3rd International Conference on Document Analysis and Recognition. 1995; 1:278–282. 10.1109/ICDAR.1995.598994

[54] Zou H, Hastie T. Regularization and variable selection via the elastic net. J R Stat Soc Ser B Stat Methodol. 2005; 67:301–20. 10.1111/j.1467-9868.2005.00503.x

[55] Fukunaga K, Narendra PM. A Branch and Bound Algorithm for Computing k-Nearest Neighbors. IEEE Trans Comput. 1975; C-24:750–53. 10.1109/T-C.1975.224297

[56] Tibshirani R. Regression Shrinkage and Selection via the Lasso. J R Stat Soc Ser B Methodol. 1996; 58:267–88. 10.1111/j.2517-6161.1996.tb02080.x

[57] Cortes C, Vapnik V. Support-vector networks. Mach Learn. 1995; 20:273–97. 10.1007/BF00994018

[58] Pedregosa F, Varoquaux G, Gramfort A, Michel V, Thirion B, Grisel O, Blondel M, Prettenhofer P, Weiss R, Dubourg V, Vanderplas J, Passos A, Cournapeau D, et al. Scikit-learn: Machine Learning in Python. J Mach Learn Res. 2011; 12:2825–30.

[59] Jaakkola T, Haussler D. Exploiting Generative Models in Discriminative Classifiers. Adv Neural Inf Process. 1999; 7.

[60] Deelen J, Uh HW, Monajemi R, van Heemst D, Thijssen PE, Böhringer S, van den Akker EB, de Craen AJ, Rivadeneira F, Uitterlinden AG, Westendorp RG, Goeman JJ, Slagboom PE, et al. Gene set analysis of GWAS data for human longevity highlights the relevance of the insulin/IGF-1 signaling and telomere maintenance pathways. Age (Dordr). 2013; 35:235–49. 10.1007/s11357-011-9340-3 22113349 PMC3543749

[61] Jeck WR, Siebold AP, Sharpless NE. Review: a meta-analysis of GWAS and age-associated diseases. Aging Cell. 2012; 11:727–31. 10.1111/j.1474-9726.2012.00871.x 22888763 PMC3444649

[62] Lu AT, Xue L, Salfati EL, Chen BH, Ferrucci L, Levy D, Joehanes R, Murabito JM, Kiel DP, Tsai PC, Yet I, Bell JT, Mangino M, et al. GWAS of epigenetic aging rates in blood reveals a critical role for TERT. Nat Commun. 2018; 9:387. 10.1038/s41467-017-02697-5 PMC5786029 29374233

[63] Archer KJ, Kimes RV. Empirical characterization of random forest variable importance measures. Comput Stat Data Anal. 2008; 52:2249–60. 10.1016/j.csda.2007.08.015

[64] Costa PT, McCrae RR, Kay G. Persons, Places, and Personality: Career Assessment Using the Revised NEO Personality Inventory. J Career Assess. 1995; 3:123–39. 10.1177/106907279500300202

[65] McCrory C, Fiorito G, Hernandez B, Polidoro S, O'Halloran AM, Hever A, Ni Cheallaigh C, Lu AT, Horvath S, Vineis P, Kenny RA. GrimAge Outperforms Other Epigenetic Clocks in the Prediction of Age-Related Clinical Phenotypes and All-Cause Mortality. J Gerontol A Biol Sci Med Sci. 2021; 76:741–49. 10.1093/gerona/glaa286 33211845 PMC8087266

[66] Marioni RE, Suderman M, Chen BH, Horvath S, Bandinelli S, Morris T, Beck S, Ferrucci L, Pedersen NL, Relton CL, Deary IJ, Hägg S. Tracking the Epigenetic Clock Across the Human Life Course: A Meta-analysis of Longitudinal Cohort Data. J Gerontol A Biol Sci Med Sci. 2019; 74:57–61. 10.1093/gerona/gly060 29718110 PMC6298183

[67] Sun ED, Michaels TCT, Mahadevan L. Optimal control of aging in complex networks. Proc Natl Acad Sci U S A. 2020; 117:20404–10. 10.1073/pnas.2006375117 32817469 PMC7456090

[68] Vural DC, Morrison G, Mahadevan L. Aging in complex interdependency networks. Phys Rev E Stat Nonlin Soft Matter Phys. 2014; 89:022811. 10.1103/PhysRevE.89.022811 25353538

[69] Stroustrup N, Anthony WE, Nash ZM, Gowda V, Gomez A, López-Moyado IF, Apfeld J, Fontana W. The temporal scaling of Caenorhabditis elegans ageing. Nature. 2016; 530:103–07. 10.1038/nature16550 26814965 PMC4828198

[70] Heidenreich PA, Trogdon JG, Khavjou OA, Butler J, Dracup K, Ezekowitz MD, Finkelstein EA, Hong Y, Johnston SC, Khera A, Lloyd-Jones DM, Nelson SA, Nichol G, et al, and American Heart Association Advocacy Coordinating Committee, and Stroke Council, and Council on Cardiovascular Radiology and Intervention, and Council on Clinical Cardiology, and Council on Epidemiology and Prevention, and Council on Arteriosclerosis, and Thrombosis and Vascular Biology, and Council on Cardiopulmonary, and Critical Care, and Perioperative and Resuscitation, and Council on Cardiovascular Nursing, and Council on the Kidney in Cardiovascular Disease, and Council on Cardiovascular Surgery and Anesthesia, and Interdisciplinary Council on Quality of Care and Outcomes Research. Forecasting the future of cardiovascular disease in the United States: a policy statement from the American Heart Association. Circulation. 2011; 123:933–44. 10.1161/CIR.0b013e31820a55f5 21262990

[71] Kaplan DT, Furman MI, Pincus SM, Ryan SM, Lipsitz LA, Goldberger AL. Aging and the complexity of cardiovascular dynamics. Biophys J. 1991; 59:945–49. 10.1016/S0006-3495(91)82309-8 2065195 PMC1281262

[72] Volpato S, Guralnik JM, Ferrucci L, Balfour J, Chaves P, Fried LP, Harris TB. Cardiovascular disease, interleukin-6, and risk of mortality in older women: the women's health and aging study. Circulation. 2001; 103:947–53. 10.1161/01.cir.103.7.947 11181468

[73] Pyrkov TV, Slipensky K, Barg M, Kondrashin A, Zhurov B, Zenin A, Pyatnitskiy M, Menshikov L, Markov S, Fedichev PO. Extracting biological age from biomedical data via deep learning: too much of a good thing? Sci Rep. 2018; 8:5210. 10.1038/s41598-018-23534-9 29581467 PMC5980076

[74] Baracho GV, Nudelman V, Isaac L. Molecular characterization of homozygous hereditary factor I deficiency. Clin Exp Immunol. 2003; 131:280–86. 10.1046/j.1365-2249.2003.02077.x 12562389 PMC1808620

[75] Fagerness JA, Maller JB, Neale BM, Reynolds RC, Daly MJ, Seddon JM. Variation near complement factor I is associated with risk of advanced AMD. Eur J Hum Genet. 2009; 17:100–04. 10.1038/ejhg.2008.140 18685559 PMC2985963

[76] Minta JO, Fung M, Paramaswara B. Transcriptional and post-transcriptional regulation of complement factor I (CFI) gene expression in Hep G2 cells by interleukin-6. Biochim Biophys Acta. 1998; 1442:286–95. 10.1016/s0167-4781(98)00189-4 9804975

[77] Anderson DH, Radeke MJ, Gallo NB, Chapin EA, Johnson PT, Curletti CR, Hancox LS, Hu J, Ebright JN, Malek G, Hauser MA, Rickman CB, Bok D, et al. The pivotal role of the complement system in aging and age-related macular degeneration: hypothesis re-visited. Prog Retin Eye Res. 2010; 29:95–112. 10.1016/j.preteyeres.2009.11.003 19961953 PMC3641842

[78] Kavanagh D, Yu Y, Schramm EC, Triebwasser M, Wagner EK, Raychaudhuri S, Daly MJ, Atkinson JP, Seddon JM. Rare genetic variants in the CFI gene are associated with advanced age-related macular degeneration and commonly result in reduced serum factor I levels. Hum Mol Genet. 2015; 24:3861–70. 10.1093/hmg/ddv091 25788521 PMC4459386

[79] Seddon JM, Yu Y, Miller EC, Reynolds R, Tan PL, Gowrisankar S, Goldstein JI, Triebwasser M, Anderson HE, Zerbib J, Kavanagh D, Souied E, Katsanis N, et al. Rare variants in CFI, C3 and C9 are associated with high risk of advanced age-related macular degeneration. Nat Genet. 2013; 45:1366–70. 10.1038/ng.2741 24036952 PMC3902040

[80] van de Ven JP, Nilsson SC, Tan PL, Buitendijk GH, Ristau T, Mohlin FC, Nabuurs SB, Schoenmaker-Koller FE, Smailhodzic D, Campochiaro PA, Zack DJ, Duvvari MR, Bakker B, et al. A functional variant in the CFI gene confers a high risk of age-related macular degeneration. Nat Genet. 2013; 45:813–17. 10.1038/ng.2640 23685748

[81] Grumach AS, Leitão MF, Arruk VG, Kirschfink M, Condino-Neto A. Recurrent infections in partial complement factor I deficiency: evaluation of three generations of a Brazilian family. Clin Exp Immunol. 2006; 143:297–304. 10.1111/j.1365-2249.2005.02988.x 16412054 PMC1809586

[82] Markiewski MM, Lambris JD. The role of complement in inflammatory diseases from behind the scenes into the spotlight. Am J Pathol. 2007; 171:715–27. 10.2353/ajpath.2007.070166 17640961 PMC1959484

[83] Schmidt JC, Cech TR. Human telomerase: biogenesis, trafficking, recruitment, and activation. Genes Dev. 2015; 29:1095–105. 10.1101/gad.263863.115 26063571 PMC4470279

[84] van de Vegte YJ, Tegegne BS, Verweij N, Snieder H, van der Harst P. Genetics and the heart rate response to exercise. Cell Mol Life Sci. 2019; 76:2391–409. 10.1007/s00018-019-03079-4 30919020 PMC6529381

[85] Blackburn EH, Epel ES, Lin J. Human telomere biology: A contributory and interactive factor in aging, disease risks, and protection. Science. 2015; 350:1193–98. 10.1126/science.aab3389 26785477

[86] Hewitt G, Jurk D, Marques FD, Correia-Melo C, Hardy T, Gackowska A, Anderson R, Taschuk M, Mann J, Passos JF. Telomeres are favoured targets of a persistent DNA damage response in ageing and stress-induced senescence. Nat Commun. 2012; 3:708. 10.1038/ncomms1708 22426229 PMC3292717

[87] GTEx Consortium. The Genotype-Tissue Expression (GTEx) project. Nat Genet. 2013; 45:580–85. 10.1038/ng.2653 23715323 PMC4010069

[88] He L, Kernogitski Y, Kulminskaya I, Loika Y, Arbeev KG, Loiko E, Bagley O, Duan M, Yashkin A, Ukraintseva SV, Kovtun M, Yashin AI, Kulminski AM. Corrigendum: Pleiotropic Meta-Analyses of Longitudinal Studies Discover Novel Genetic Variants Associated with Age-Related Diseases. Front Genet. 2018; 8:226. 10.3389/fgene.2017.00226 29375618 PMC5771150

[89] Grammatikakis I, Panda AC, Abdelmohsen K, Gorospe M. Long noncoding RNAs(lncRNAs) and the molecular hallmarks of aging. Aging (Albany NY). 2014; 6:992–1009. 10.18632/aging.100710 25543668 PMC4298369

[90] Greco S, Gorospe M, Martelli F. Noncoding RNA in age-related cardiovascular diseases. J Mol Cell Cardiol. 2015; 83:142–55. 10.1016/j.yjmcc.2015.01.011 25640162 PMC5509469

[91] Kim C, Kang D, Lee EK, Lee JS. Long Noncoding RNAs and RNA-Binding Proteins in Oxidative Stress, Cellular Senescence, and Age-Related Diseases. Oxidative Medicine and Cellular Longevity. 2017. https://www.hindawi.com/journals/omcl/2017/2062384/abs/'>https://www.hindawi.com/journals/omcl/2017/2062384/abs/10.1155/2017/2062384 PMC5547732 28811863

[92] Kour S, Rath PC. Long noncoding RNAs in aging and age-related diseases. Ageing Res Rev. 2016; 26:1–21. 10.1016/j.arr.2015.12.001 26655093

[93] Chen T, Guestrin C. XGBoost: A Scalable Tree Boosting System. KDD '16: Proceedings of the 22nd ACM SIGKDD International Conference on Knowledge Discovery and Data Mining. 2016:785-94. 10.1145/2939672.2939785

[94] Glass D, Viñuela A, Davies MN, Ramasamy A, Parts L, Knowles D, Brown AA, Hedman AK, Small KS, Buil A, Grundberg E, Nica AC, Di Meglio P, et al, and UK Brain Expression consortium, and MuTHER consortium. Gene expression changes with age in skin, adipose tissue, blood and brain. Genome Biol. 2013; 14:R75. 10.1186/gb-2013-14-7-r75 23889843 PMC4054017

[95] Peters MJ, Joehanes R, Pilling LC, Schurmann C, Conneely KN, Powell J, Reinmaa E, Sutphin GL, Zhernakova A, Schramm K, Wilson YA, Kobes S, Tukiainen T, et al, and NABEC/UKBEC Consortium. The transcriptional landscape of age in human peripheral blood. Nat Commun. 2015; 6:8570. 10.1038/ncomms9570 26490707 PMC4639797

