# Supplementary material for: Predicting physiological aging rates from a range of quantitative traits using machine learning
Source: Aging (Albany NY). 2021 Oct 29;13(20):23471–516. doi: 10.18632/aging.203660 (PMC8580337; doi:10.18632/aging.203660)
Supplement: Supplementary Methods [file aging-13-203660-s001.pdf]

SUPPLEMENTARY METHODS

Description of data cleaning and binning procedures

Dataset cleaning strategy

Both SardiNIA and InCHIANTI datasets contained substantial portions of missing values. Traits and samples were dropped iteratively to preserve maximum data size while maintaining a complete set of values. In wave 1 of SardiNIA, the cleaning method reduced the number of participants from 6165 to 4817 and the number of traits from 183 to 148.

Binning strategy

Binning strategies were determined empirically to maximize both model performance and data retention. We abbreviated a binning strategy as a tuple (bin size, youngest age, oldest age). In the SardiNIA dataset, optimal binning for wave 1 was set at (5, 12, 77), which allowed for 120 training samples and 13 testing samples from each bin per split and 13 total age bins (1560 training and 169 test samples in total). For wave 2, bins with (5, 16, 81) were determined and yielded 83 training and nine testing samples per age bin with 13 total bins (1079 training and 117 test samples in total). For wave 3, bins with (5, 20, 75) were determined and yielded 81 training and eight testing samples per age bin with 12 total bins (984 training and 96 test samples in total). For wave 4, bins with (5, 21, 81) were determined and yielded 49 training and five testing samples for each of 12 age bins (588 training and 60 testing samples in total). A schematic of this binning is shown in Supplementary Figure 2.

We used different binning strategies for the common-trait framework in SardiNIA to maximize sample representation. The same age ranges and bin sizes (5 years) were used to make fair comparisons to the full-trait model. Sample sizes were increased to saturate the smallest bin size: wave 1 (160 training, 18 testing), wave 2 (107 training, 12 testing), wave 3 (213 training, 24 testing), wave 4 (64 training, seven testing).

As a study of frailty in older individuals, InCHIANTI contained much lower representation of younger age groups. Despite this, the same binning strategy was employed to maintain fair comparisons to SardiNIA. Interestingly, the smaller sample sizes did not substantially decrease model performance. In the baseline study (wave 0), bins of (6, 23, 89) provided sufficient data for training (19) and testing (2) for each of 11 age bins (209 training and 22 testing samples in total). For wave 1, we used (6, 26, 86) which yielded 19 training and two testing samples for each of 10 age bins (190 training and 20 testing in total). For wave 2, we used (6, 27, 87) which yielded 19 training and two testing samples for each of 10 age bins (190 training and 20 testing in total). For wave 3, we used (6, 31, 91) which yielded 20 training and two testing samples for each of 10 age bins (200 training and 20 testing in total).

Machine learning model selection

Description of main machine learning models investigated in the study

In addition to the random forest classifier, we explored other classical machine learning models outlined below.

Elastic net

We utilized the Scikit-Learn implementation of elastic net regression (ElasticNet) [58], which was equivalent to the glmnet implementation in R with “alpha” and “l1_ratio” in Scikit-Learn corresponding to “lambda” and “alpha” in glmnet. Elastic net uses a penalty defined by a linear combination of the L1 and L2 penalties of the LASSO and Ridge regressions. In our model, we used the default uniform penalty weights specified by “l1_ratio = 0.5” and “alpha = 1.0”.

K-nearest neighbors

We implemented the k-nearest neighbors regressor from the Scikit-Learn library [58]. The algorithm predicts the target variable from local interpolation of the k nearest neighboring data points in high-dimensional trait space. Our model used “n_neighbors = 20”, “weights = 'distance'”, “*p* = 1”, which were determined heuristically.

Support vector machine (SVR)

We implemented the epsilon-support vector regressor (SVR) from the Scikit-Learn library [58]. Our model used the default parameters of “C = 1.0” and “epsilon = 0.1”.

Multiple linear regression (LinReg)

We implemented the default multiple linear regression model (LinearRegression) from the Scikit-Learn library [58].

Lasso

Lasso regression was implemented from the Scikit-Learn library [58]. We used the elastic net model (ElasticNet) with “l1_ratio = 1.0” to recover the lasso regression.

Saturation of model performance from traits and samples

Model performance measured with R^2 saturated after an intermediate number of traits and after an intermediate

number of individuals (i.e., the saturation in performance occurred before the maximum number of traits or individuals was reached in virtually all cases). For example, increasing the number of traits resulted in marginal R^2 gains after ~30 traits and after ~80 individuals per bin in the RFC full-trait model for the SardiNIA baseline study (Supplementary Figure 3). Corroborating these results, we observed no significant difference in model performance (R^2) when the model was trained on the entire data as compared to uniform sampling from age bins (data not shown).

Trait transformation with linear discriminant analysis (LDA) prior to training/testing increased predictive performance in most machine learning models (Supplementary Figure 3).

Physiological age and physiological aging rate (PAR) computed with different model frameworks

Random forest classifier (RFC) model for follow-up waves

Model performances for the three follow-up studies (W2, W3, W4) were highly similar to that of the baseline (W1) SardiNIA study (see Supplementary Figure 4). Physiological ages were well-correlated with chronological age, and the PAR values were approximately uniformly distributed and weakly correlated with chronological age. It should be noted that model performance (R^2) decreased for the later follow-up studies, which was likely due to a reduced number of participants and reduced overlap with the original trait measurements in the W1.

InCHIANTI-trained random forest classifer model (RFC)

We replicated our findings in the InCHIANTI longitudinal study. The same RFC machine learning framework yielded comparably well-correlated physiological age measurements as those from the SardiNIA study (see Supplementary Figure 5). Likewise, the distribution of PAR measurements was roughly uniform with age across all waves of the InCHIANTI study.

Similar distribution of PARs observed in SardiNIA and InCHIANTI studies

Despite training the RFC models on studies with different sets of quantitative traits, the PARs of individuals in the SardiNIA study and the PARs of individuals in the InCHIANTI were distributed similarly (see Supplementary Figure 7A). Furthermore, most of the difference in PAR distribution between the two studies are explained by the oldest and youngest age bins. By removing PAR predictions for these edge bins, the PAR distributions are nearly identical for SardiNIA and InCHIANTI (Supplementary Figure 7B).

Common-trait model (RFC) trained on SardiNIA data

Reducing the traits to a subset of common clinical and cardiovascular measurements from the SardiNIA study (see Supplementary Table 1 for the description of common traits) resulted in comparable model performance as in the full-trait model. Physiological ages were well-correlated with chronological age, and PARs were weakly correlated with chronological age across all SardiNIA studies. Similarly, the later follow-up waves observed lower model performance, possibly due to decreased numbers of participants and overlapping traits. The PARs obtained from the full-trait model and the common-trait model were highly correlated (R^2 = 0.798; see Supplementary Figure 9).

Elastic net regression model trained on SardiNIA data

A high-performing regression method was the elastic net regressor (R^2 = 0.84 for SardiNIA W1). Previous studies have used elastic net regression for age estimation from blood biochemical measurements [41] and for DNAm age calculation [1, 20]. Using the elastic net model in lieu of the random forest model yielded comparable results across each baseline and follow-up study of SardiNIA (data not shown).

Gender-separated analysis of PARs

To determine if physiological differences between the sexes biased the predictive models, we analyzed the PAR measurements from the RFC model developed on data including both genders to determine any differences in the mean or spread of the PARs. We observed no significant difference in the physiological aging rate (PAR) and physiological age acceleration (PAA) between male and female study participants (Supplementary Figure 6).

Top traits in SardiNIA and InCHIANTI

Top traits for the RFC model in InCHIANTI study

The top traits in the InCHIANTI study were obtained using baseline data with the same two scoring strategies. Several cardiovascular traits were highly ranked including pulse wave velocity (VEL), systolic blood pressured (23_V28), proximal amplitude (APROX), repolarization phase (RIPOL), and atrioventricular conduction time (TAV) among others; which reflected the high ranking of cardiovascular traits in the SardiNIA data. Another top trait across multiple ranking methods was creatinine clearance (CLCR, UCRE24), which is an important indicator of renal health (Cockcroft & Gault, 1976) and has been

associated with aging (Anderson & Brenner, 1986). Creatinine levels were also highly ranked in SardiNIA. Additional overlaps between top 20% InCHIANTI and the highest-ranking SardiNIA traits include waist circumference (VITA), blood nitrogen levels (BUN), blood fibrinogen levels (FIBRIN), cholesterol levels (COLTOT, COLLDL, OX_LDL), sodium intake (VN26), hypertension (IPERT1), uric acid (URICO), transferrin (STRMG), IL-6 (IL_6). Other top traits corresponded to frailty markers such as general frailty (FRAIL, ALLFRA, NALLFR), exhaustion (FEXHAU), weight loss (FWGTLS), grip strength (FSTRNC), visual acuity (24_V35), and gait speed (WLK1A, WSPD1B, WLK1MN, 17_V2, FWLKCT)—many of which have been linked to aging (Fulop et al., 2010; Kan et al., 2010; Bohannon et al., 2008). Other high-ranking traits from the three scoring methods included diagnosis of dementia (VASDEM, DEMENT), insulin-like growth factor I (TIGF), fatty acid levels (TFA_MO, C24_1B), coordination (PEGONE, 25_V14, 22_V34), and a variety of coordination task and questionnaire results (18_V4, 18_V8, 25_V24, 25_V13, 4_V75, 5_V26, 1_N8, 17_V2, 8_V1, 12_V1, Q0208, etc). Refer to Supplementary Figure 16.

Top traits for the common-trait RFC model in SardiNIA

The top traits among the common clinical and cardiovascular SardiNIA traits were determined using the three scoring methods outlined in the Methods section. For these methods, most of the top traits from the full-trait set were present. The overlapping traits included pulse wave velocity (pwv), CCA intima media thickness (vasIMT), other cardiovascular traits (vasEDV, vasvti, vasPSV, vasIP, vasDiaDiam, etc), and waist circumference (exmWaist). Additional overlaps included various blood serum levels of sodium (labsSodiedemia), uric acid (labsAcidourico), and alanine aminotransferase (labsALT). A notable difference in the top traits for the common-trait model was the lack of NEOPIR personality traits (o1, e5, a4, form), which were not present in the common-trait set. There was high rank-correlation between the common-trait and full-trait sets (Spearman correlation, $\rho = 0.92$). Refer to Supplementary Figure 15.

Reproducibility of PAR measurements across follow-up studies

RFC model in SardiNIA study

We determined the reproducibility of individual PARs across time by using the RFC model to measure PARs across all four waves of the SardiNIA study. The correlation between the PARs across each consecutive wave (Δt = 3–4 years) was relatively stable (e.g., $R^2 \sim 0.4$ between the first two waves, see Supplementary Figure 18) but decayed over longer time periods (e.g., $R^2 \sim 0.3$ for Δt = 6–7 years, $R^2 \sim 0.2$ for Δt = 9–10 years). PARs derived from the common-trait model observed the same trends with marginally higher temporal stability (see following subsection). Notably, the InCHIANTI PARs showed higher consistency over time than those from SardiNIA ($R^2 = 0.55$ between baseline and follow-up #1, $R^2 = 0.52$ between baseline and follow-up #2, $R^2 = 0.46$ between baseline and follow-up #3, see following subsection). These results indicated that the physiological aging rate was stable for an individual in the short-term—from several years up to a decade—but appreciably destabilized over longer periods of time. This destabilization may be reflecting the environmental influences and heterogeneities. PAR trajectories showcased the generally observed trend in which slow agers retained low PARs while fast agers retained high PARs across time (see Supplementary Figure 18A, 18B). In fact, the average standard deviation between PARs for a given individual across the four waves was around 0.1 for both the full-trait model and the common-trait model.

Common-trait RFC model in SardiNIA study

PAR measurements obtained using the common-trait model for the same individual were correlated across each of the baseline and follow-up studies of SardiNIA (see Supplementary Figure 18C, 18D) such that there was notable stability of PAR estimates over the period of a few years. The mean standard deviation of individual PAR measurements across the four waves of the study was 0.085. The correlation between PARs decreased monotonically with increased time (Δt) between the measurements, which suggested that environmental influences can noticeably change the PAR after the span of a few years. Alternatively, this destabilization in the PAR values over longer time periods may represent an artefact of changes in data collection procedures and trait measurements in the longitudinal studies.

RFC model in InCHIANTI study

PAR measurements obtained from the RFC model for the same individual were highly correlated across each of the baseline and follow-up studies of InCHIANTI (see Supplementary Figure 18E, 18F) such that there was notable stability of PAR estimates over the period of a few years. The mean standard deviation of individual PAR measurements across the four waves of the study was 0.048. The correlation between PARs appeared to decrease with increased time (Δt) between the measurements, which corroborated similar observations made in the SardiNIA models.

Alternative age acceleration metric

Physiological age acceleration (PAA)

The physiological age acceleration (PAA) corresponds to the difference between the physiological age and chronological age of an individual and was calculated as

$$PAA = Physiological\ Age - Chronological\ Age$$

The PAA is similar to the PAR in that it measures the progression of an individual's aging trajectory relative to other individuals with the same chronological age. This measurement was most comparable to DNAm age acceleration.

Epigenetic age acceleration (EAA)

The epigenetic age acceleration was similarly calculated as the difference between the predicted epigenetic age and the chronological age:

$$EAA = Epigenetic\ Age - Chronological\ Age$$

Our measurement of epigenetic age acceleration was identical to previous methods of calculating intrinsic epigenetic age acceleration [62].

Controlling for sex and age in the comparison of PAR and EAR

In the main text, we presented the correlation between PAR and EAR without adjusting for any co-variation by sex or chronological age (see Figure 3). Since both aging rates are likely to be at least partially dependent on chronological age (and sex to an extent), we also compared the residual PAR and EAR values after regressing out sex and chronological age using an ordinary least-squares linear model. The correlation between PAR and EAR was reduced but still positive (R^2 = 0.182; see Supplementary Figure 12).

Linear rescaling and trimming to correct for the imbalanced distribution of PARs across age

Motivation for linearly rescaled predicted ages

The random forest classifier showcased a slight deviation from a uniform distribution of PAR measurements despite the high predictive performance (R^2 = 0.86 between physiological age and chronological age). Due to the deviation, the oldest individuals recorded mean PAR < 1 and the youngest individuals had mean PAR > 1. The deviation may be representative of selection bias from the assumedly longer survival of slower aging (PAR < 1) individuals. However, another possible source of this deviation was the lack of data for individuals beyond the age range specified in SardiNIA. The elastic net model, a regression method, produced a notably milder deviation. To reduce the deviation, we attempted to linearly rescale the predicted ages into the same dimensions as chronological age and enforce a slope of ~1.0 between the rescaled physiological age and the chronological age (see Supplementary Figure 14A). The rescaling preserved the relative information obtained from the predictive model and reduced age-associated imbalances in the PAR. The rescaling resulted in normal distributions of PARs centered around a mean age value corresponding to the chronological age of that group (see Supplementary Figure 14B). The equation for linear rescaling was

$$[Physiological\ Age] = \frac{[Predicted\ Age] - \beta}{\alpha},$$

where α was the slope of the linear least-squares regression with an L2 norm on coordinate pairs determined by (chronological age, predicted age), and β was the corresponding intercept. The L2 norm was used since we expected the physiological ages to be normally distributed around the chronological age for all individuals of that given chronological age.

Analytic details for the linear rescaling of physiological age

A linear rescaling was applied to the predicted ages to obtain the physiological age measurements. The rescaling enforced the mean of the physiological ages as the chronological age corresponding to each age group and therefore symmetrized the distribution of the eRAs across all ages. This was achieved with:

$$[Physiological\ Age] = \frac{[Predicted\ Age] - \beta}{\alpha}$$

Where α was the slope of the linear least-squares regression with an L2 norm on coordinate pairs determined by (chronological age, predicted age), and β was the corresponding intercept. The L2 norm was used since we expect the physiological ages to be normally distributed around the chronological age for all individuals of that given chronological age.

In this section, we provide an informal sketch for why the rescaling equation results in the intended centering of the physiological ages around the chronological age. We denote the chronological age as x and the predicted (unscaled) age as y_1. Using a linear least squares regression with an L2 norm, we obtain the linear model parameters α and β such that the line that minimizes the L2 residual is defined as:

$$y_1^{ls} = \alpha x + \beta$$

We apply the rescaling transformation to obtain the physiological (rescaled) age denoted y_2. Similarly, we can evaluate the linear least squares regression on the rescaled data to obtain linear model parameters that minimize the L2 norm such that:

$$\alpha_2, \beta_2 = \underset{\alpha_2, \beta_2}{\text{argmin}} \sum_i \left| y_{2,i}^{ls} - y_{2,i} \right|^2$$
$$y_{2,i}^{ls} = \alpha_2 x + \beta_2$$

Substituting the definition of $y_{2,i}^{ls}$ into the objective function yields:

$$\alpha_2, \beta_2 = \underset{\alpha_2, \beta_2}{\text{argmin}} \sum_i \left| \alpha_2 x + \beta_2 - y_{2,i} \right|^2$$

Since $y_{2,i}$ is the rescaled predicted age, then it must be that $y_{2,i} = \frac{y_{1,i} - \beta}{\alpha}$. Additionally, we can rewrite $x = \frac{y_1^{ls} - \beta}{\alpha}$. Substituting in these two relations yields:

$$\alpha_2, \beta_2 = \underset{\alpha_2, \beta_2}{\text{argmin}} \sum_i \left| \alpha_2 \frac{y_1^{ls} - \beta}{\alpha} + \beta_2 - \frac{y_{1,i} - \beta}{\alpha} \right|^2$$
$$= \underset{\alpha_2, \beta_2}{\text{argmin}} \sum_i \left| \frac{1}{\alpha}(\alpha_2 y_1^{ls} - y_{1,i}) - \frac{\beta}{\alpha}(\alpha_2 - 1) + \beta_2 \right|^2$$

This is minimized for values of $\alpha_2 = 1,\ \beta_2 = 0$ where it reduces the physiological age objective function to

$$\sum_i \left| \frac{y_1^{ls} - y_{1,i}}{\alpha} \right|^2 = \frac{1}{\alpha^2} \sum_i \left| y_1^{ls} - y_{1,i} \right|^2$$

which is the minimal objective value for the predicted (unscaled) ages. As a result, the least squares linear regression for the rescaled physiological ages can be approximated as a rescaling of the linear regression for the unscaled predicted ages.

Description of trimmed PAR values

We implemented another approach to reduce the age-associated imbalance in PAR measurements, which was to include the youngest and oldest edge bins (first five years and last five years for SardiNIA W1) in model training but exclude them from downstream analyses. We refer to the reduced set of measurements as the "trimmed" physiological age and "trimmed" physiological aging rate. Trimming increased the slope of the linear regression between chronological age and physiological age.

Additional details on GWAS significant loci

APLF (p = 8.59E-8) encodes a histone chaperone protein that is involved in non-homologous end-joining (NHEJ) repair of DNA double strand breaks [96, 97], which is linked to aging and age-related disease [98]. ARHGAP15 is associated with diverticulitis [99] and colorectal cancer [100], and was selectively up-regulated with age (r = 0.294) in colon tissue according to GTEx RNA-seq gene expression profiles. ANKRD26 (p = 2.96E-7) has been associated with human diabetes mellitus, cardiovascular disease, and neurodegenerative disease in previous genome-wide studies [88] and has been shown to promote diabetes and obesity in mouse models [101, 102]. ANKRD26 is flanked by the LINC00202 locus (see Figure 4A), but analysis with GTEx [87] did not identify any of the significant LINC00202 SNPs as eQTLs for ANKRD26. ZNF518B (p = 3.26E-7) has been associated with gout [103], colorectal tumor invasion [104, 105], age-related epigenetic changes [106], and is involved in histone modification [107]. Notably, ZNF518B is down-expressed with age in all non-brain GTEx tissues examined including heart (r = −0.231), liver (r = −0.475), lung (r = −0.294), thyroid (r = −0.405), and colon (r = −0.250) (see Supplementary Materials). CSMD1 (p = 3.96E-7) was previously associated with familial Parkinson's disease [108] and cognitive function [109]. Accordingly, CSMD1 was down-expressed with age (r = −0.314) in GTEx cerebellum samples and did not appear to be age-associated in the non-brain tissues examined. Using GTEx data, we generated plots of the normalized gene expression value across age for several key genes of interest (see Supplementary Figure 19). Normalized gene expression value was computed as the sum-normalized value for all gene expression values in a given sample/patient (similarly to TPM calculations).

Common-trait physiological aging rates predict mortality

To determine whether common-trait PARs predicted mortality and lifespan, we performed a random one-to-one age-matched comparison on the 329 deceased participants and the remaining living participants in the SardiNIA study. The difference in the mean PAR measurements of the two groups was calculated as $\Delta PAR = PAR_{deceased} - PAR_{living}$, and a corresponding p-value was obtained from a one-sided ($\Delta PAR > 0$) one-sample t-test. The age-matched grouping was performed 10000 times and ΔPAR and p-values. The fraction of significantly different ($p < 0.05$) mean PAR values between the deceased and living groups was 65.7% and the mean ΔPAR was 0.013 (see Supplementary Figure 11). More than 99% of the 10000 random age-matched comparisons reported $\Delta PAR > 0$ as compared to randomized controls where 50.8% of the comparisons between two randomly age-matched groups had $\Delta PAR > 0$ (Supplementary Figure 11). Estimated lifespans were similarly negatively correlated with PARs in the common-trait model (r = −0.469, Supplementary Figure 11).

Computational pipeline and code repository

The computational pipelines can be accessed at the Github code repository: https://github.com/sunericd/SardiNIAgeRates. The repository contains two Python scripts ("runModel.py" and "AgeRatesTurnkey.py") that are necessary for

running the machine learning framework. The model parameters are specified in the "run_spec.txt" file. In the working directory, the machine learning framework can be run with the command line python runModel.py. After successfully running the model, results will be saved as image files and a tab-separated text file with columns corresponding to [ID] [Age] [Predicted Age] [Physiological Aging Rate] and the rows corresponding to each individual in the SardiNIA/InCHIANTI datasets.

The Github repository also contains two Jupyter notebooks ("results_and_analyses.ipynb" and "top_traits.ipynb") that outline code blocks for generating most of the figures included in the main text and supplementary materials and other examples for analyzing the results of the machine learning framework. Additional Jupyter notebooks in the "Miscellaneous" directory on the repository contain code used to test and develop the predictive frameworks outlined in this investigation.

The computational model was written in Python 3.5 and requires NumPy, SciPy, Matplotlib, Pandas, and Scikit-learn to be installed. Optionally, XGBoost can be installed.

REFERENCES

96. Mehrotra PV, Ahel D, Ryan DP, Weston R, Wiechens N, Kraehenbuehl R, Owen-Hughes T, Ahel I. DNA repair factor APLF is a histone chaperone. Mol Cell. 2011; 41:46–55. https://doi.org/10.1016/j.molcel.2010.12.008 PMID:21211722

97. Rulten SL, Fisher AE, Robert I, Zuma MC, Rouleau M, Ju L, Poirier G, Reina-San-Martin B, Caldecott KW. PARP-3 and APLF function together to accelerate nonhomologous end-joining. Mol Cell. 2011; 41:33–45. https://doi.org/10.1016/j.molcel.2010.12.006 PMID:21211721

98. Lombard DB, Chua KF, Mostoslavsky R, Franco S, Gostissa M, Alt FW. DNA repair, genome stability, and aging. Cell. 2005; 120:497–512. https://doi.org/10.1016/j.cell.2005.01.028 PMID:15734682

99. Sigurdsson S, Alexandersson KF, Sulem P, Feenstra B, Gudmundsdottir S, Halldorsson GH, Olafsson S, Sigurdsson A, Rafnar T, Thorgeirsson T, Sørensen E, Nordholm-Carstensen A, Burcharth J, et al. Sequence variants in ARHGAP15, COLQ and FAM155A associate with diverticular disease and diverticulitis. Nat Commun. 2017; 8:15789. https://doi.org/10.1038/ncomms15789 PMID:28585551

100. Pan S, Deng Y, Fu J, Zhang Y, Zhang Z, Ru X, Qin X. Decreased expression of ARHGAP15 promotes the development of colorectal cancer through PTEN/AKT/FOXO1 axis. Cell Death Dis. 2018; 9:673. https://doi.org/10.1038/s41419-018-0707-6 PMID:29867200

101. Bera TK, Liu XF, Yamada M, Gavrilova O, Mezey E, Tessarollo L, Anver M, Hahn Y, Lee B, Pastan I. A model for obesity and gigantism due to disruption of the Ankrd26 gene. Proc Natl Acad Sci U S A. 2008; 105:270–75. https://doi.org/10.1073/pnas.0710978105 PMID:18162531

102. Raciti GA, Bera TK, Gavrilova O, Pastan I. Partial inactivation of Ankrd26 causes diabetes with enhanced insulin responsiveness of adipose tissue in mice. Diabetologia. 2011; 54:2911–22. https://doi.org/10.1007/s00125-011-2263-9 PMID:21842266

103. Jin TB, Ren Y, Shi X, Jiri M, He N, Feng T, Yuan D, Kang L. Genetic variations in the CLNK gene and ZNF518B gene are associated with gout in case-control sample sets. Rheumatol Int. 2015; 35:1141–47. https://doi.org/10.1007/s00296-015-3215-3 PMID:25591661

104. Gimeno-Valiente F, Riffo-Campos ÁL, Vallet-Sánchez A, Siscar-Lewin S, Gambardella V, Tarazona N, Cervantes A, Franco L, Castillo J, López-Rodas G. ZNF518B gene up-regulation promotes dissemination of tumour cells and is governed by epigenetic mechanisms in colorectal cancer. Sci Rep. 2019; 9:9339. https://doi.org/10.1038/s41598-019-45411-9 PMID:31249328

105. Valiente FG, Riffo-Campos AL, Cervantes A, López-Rodas G, Franco L, Castillo J. First report of ZNF518B gene expression as a prognostic factor in colorectal cancer development: Role in tissue invasiveness. Ann Oncol. 2018; 29. https://doi.org/10.1093/annonc/mdy314.034

106. Bacos K, Gillberg L, Volkov P, Olsson AH, Hansen T, Pedersen O, Gjesing AP, Eiberg H, Tuomi T, Almgren P, Groop L, Eliasson L, Vaag A, et al. Blood-based biomarkers of age-associated epigenetic changes in human islets associate with insulin secretion and diabetes. Nat Commun. 2016; 7:11089. https://doi.org/10.1038/ncomms11089 PMID:27029739

107. Maier VK, Feeney CM, Taylor JE, Creech AL, Qiao JW, Szanto A, Das PP, Chevrier N, Cifuentes-Rojas C, Orkin SH, Carr SA, Jaffe JD, Mertins P, Lee JT. Functional Proteomic Analysis of Repressive

Histone Methyltransferase Complexes Reveals ZNF518B as a G9A Regulator. Mol Cell Proteomics. 2015; 14:1435–46. https://doi.org/10.1074/mcp.M114.044586 PMID:25680957

108. Ruiz-Martínez J, Azcona LJ, Bergareche A, Martí-Massó JF, Paisán-Ruiz C. Whole-exome sequencing associates novel *CSMD1* gene mutations with familial Parkinson disease. Neurol Genet. 2017; 3:e177. https://doi.org/10.1212/NXG.0000000000000177 PMID:28808687

109. Athanasiu L, Giddaluru S, Fernandes C, Christoforou A, Reinvang I, Lundervold AJ, Nilsson LG, Kauppi K, Adolfsson R, Eriksson E, Sundet K, Djurovic S, Espeseth T, et al. A genetic association study of CSMD1 and CSMD2 with cognitive function. Brain Behav Immun. 2017; 61:209–16. https://doi.org/10.1016/j.bbi.2016.11.026 PMID:27890662
