# Supplementary material for: Predicting physiological aging rates from a range of quantitative traits using machine learning
Source: Aging (Albany NY). 2021 Oct 29;13(20):23471–516. doi: 10.18632/aging.203660 (PMC8580337; doi:10.18632/aging.203660)
Supplement: Supplementary Figures [file aging-13-203660-s002.pdf]

## SUPPLEMENTARY FIGURES

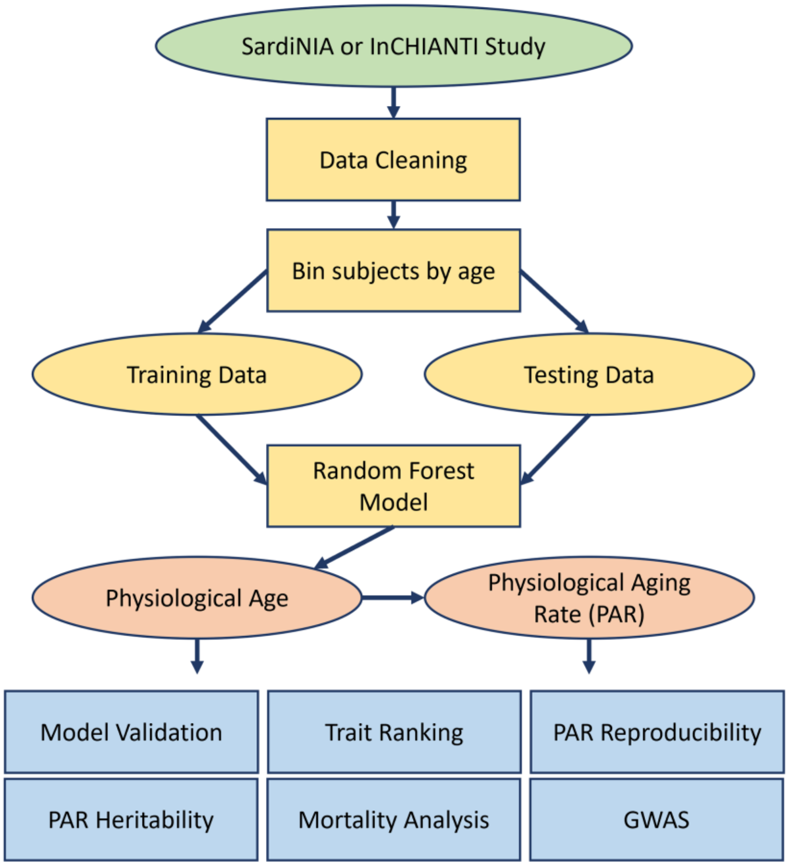


**Supplementary Figure 1. Computational workflow for measuring physiological age and physiological aging rates (PAR) using the machine learning framework.** These quantities were used to determine the most informative traits from the SardiNIA and InCHIANTI physiological, cognitive, and molecular markers. We measured the reproducibility and heritability of PARs. PARs from deceased participants were compared to age-matched living participants to determine the association of PAR with mortality and survival. GWAS on the PARs revealed significant genetic loci associated with differences in the aging rate.

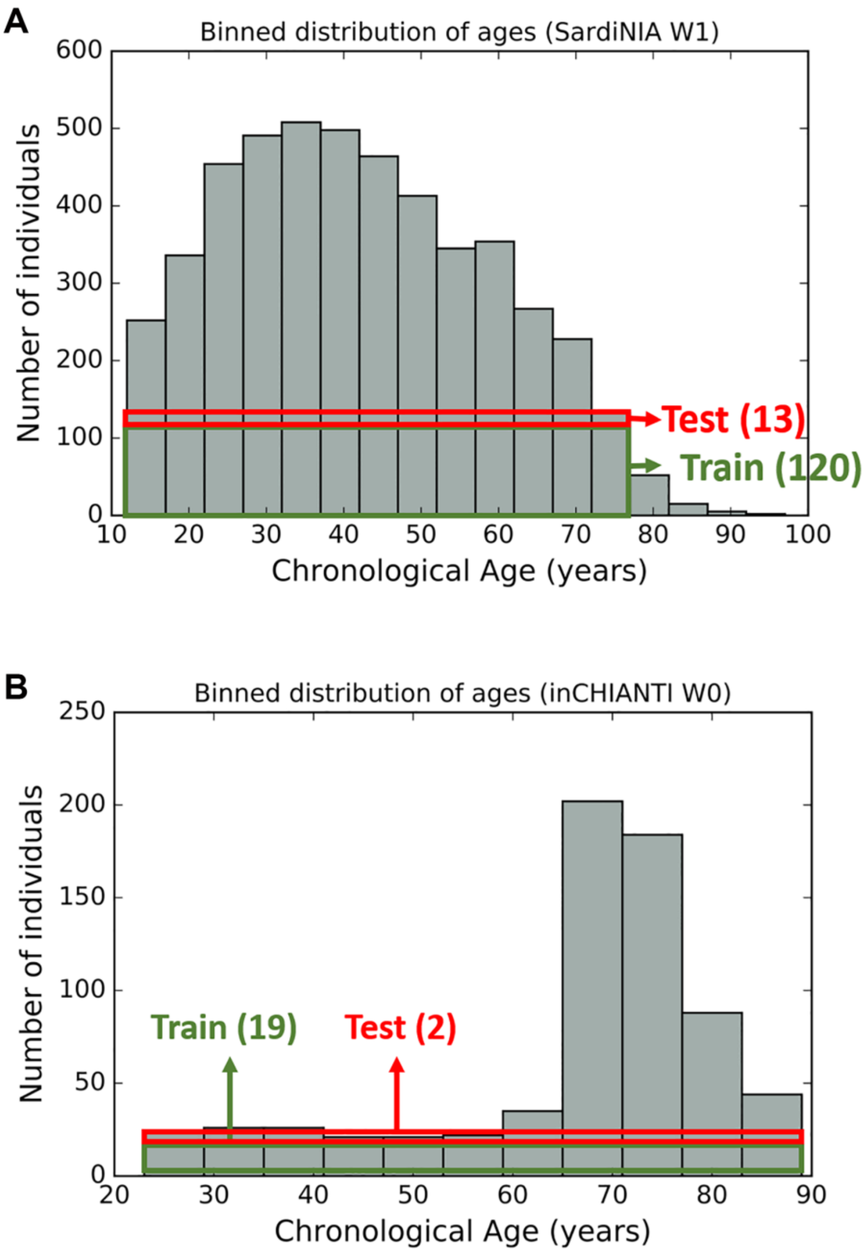


**Supplementary Figure 2. Age bin distributions for the baseline SardiNIA and InCHIANTI data.** Training (green) and testing (red) sample sizes per bin are labelled for the baseline study figures in each dataset. (**A**) In the baseline study of SardiNIA (W1), the individuals were binned into 5-year age groups spanning 12 years to 77 years of age. For each random training and testing set split, each bin contributes 120 training samples and 13 testing samples, resulting in a total set of 1560 training and 169 testing samples. (**B**) The baseline InCHIANTI study (W0) had 19 training and 2 testing samples per age bin (11 total bins), which resulted in a total of 209 training samples and 22 testing samples per training-testing split. Similar binning strategies for the follow-up studies and SardiNIA common-trait model are outlined in the text.

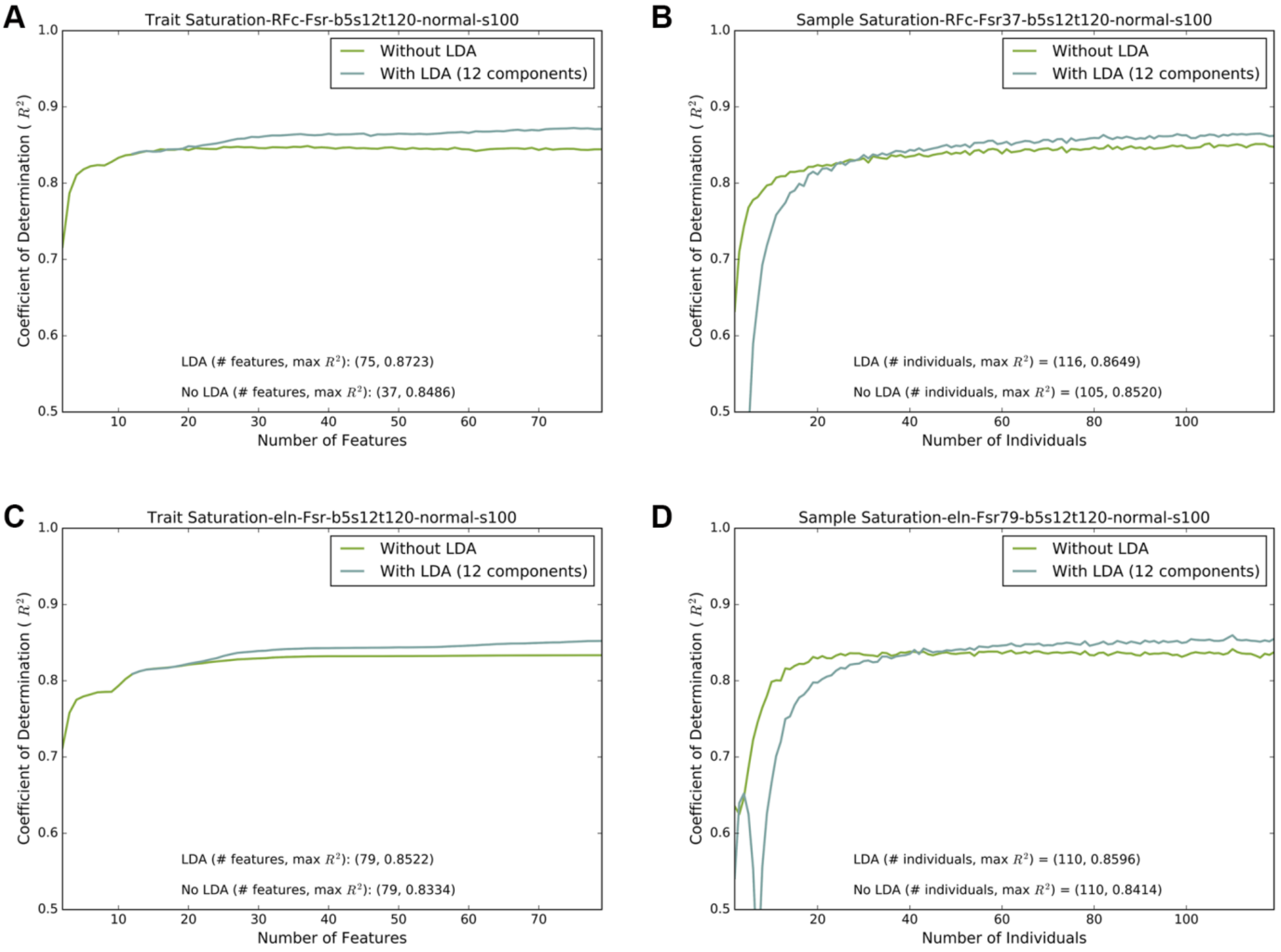


**Supplementary Figure 3. Model performance saturates for increasing number of rank-ordered traits and samples.** Trends for the two main machine learning models (RFC, ElNet) are depicted with or without trait transformation by linear discriminant analysis (LDA). LDA improved model performance in virtually all cases. (**A**) Predictive performance ($R^2$) of RFC saturated around 30 rank-ordered features when using all 120 individuals, and (**B**) saturated around 80 individuals per bin when using 37 rank-ordered features. (**C**) Predictive performance ($R^2$) of ElNet saturated around 60 rank-ordered features when using all 120 individuals, and (**D**) saturated around 50 individuals per bin when using 79 rank-ordered features. Model performance typically saturated before the maximum number of traits or samples was reached. Results were averaged over 100 training-testing splits for the baseline SardiNIA study.

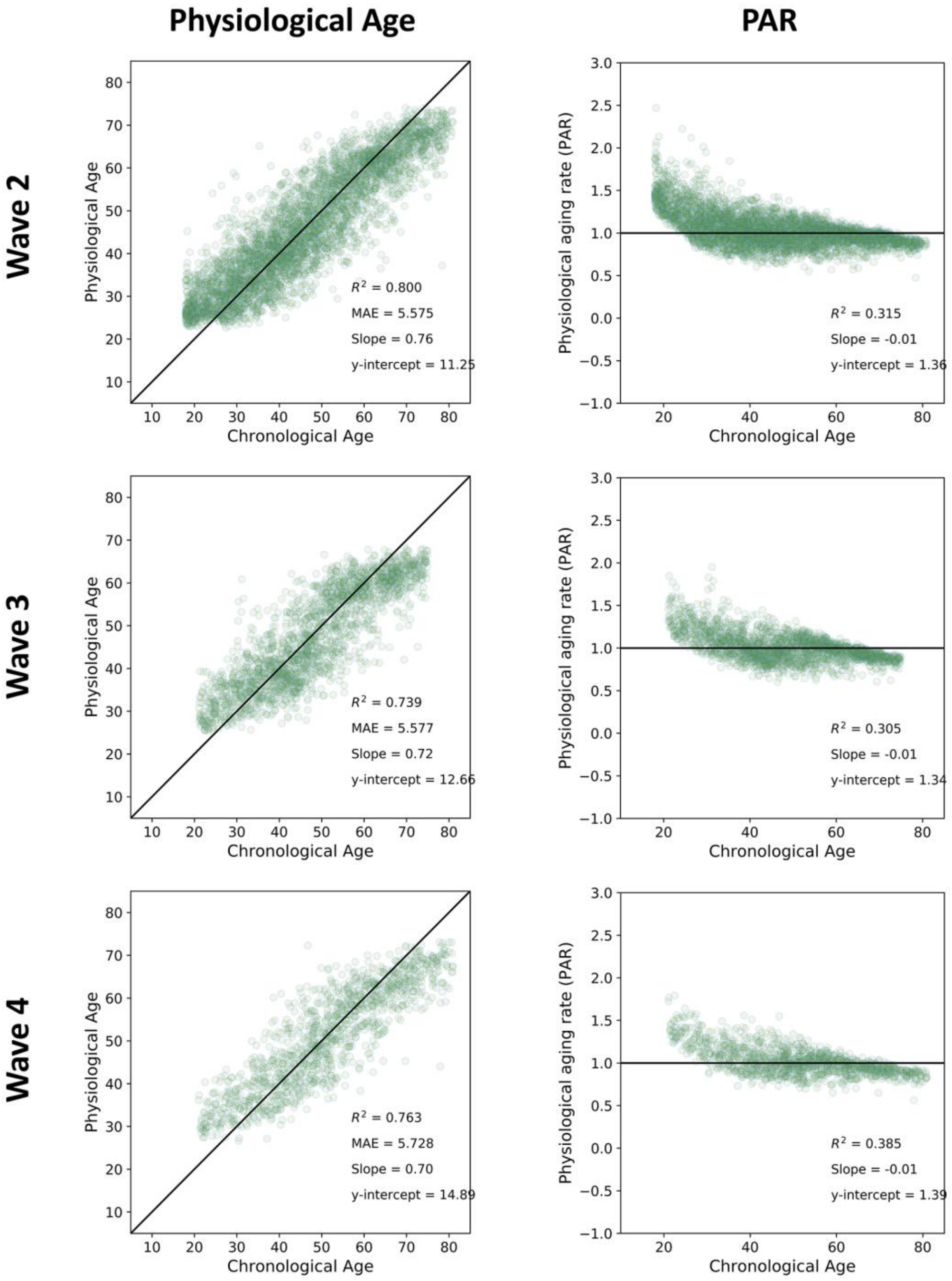


**Supplementary Figure 4. Plots of physiological age and PAR against chronological age for all follow-up studies of SardiNIA using the full-trait RFC model.** Physiological wages were well-correlated to chronological age while PAR measurements were weakly correlated with chronological age across all follow-up studies. Figures for the baseline SardiNIA study (W1) are shown in Figure 2C of the main text.

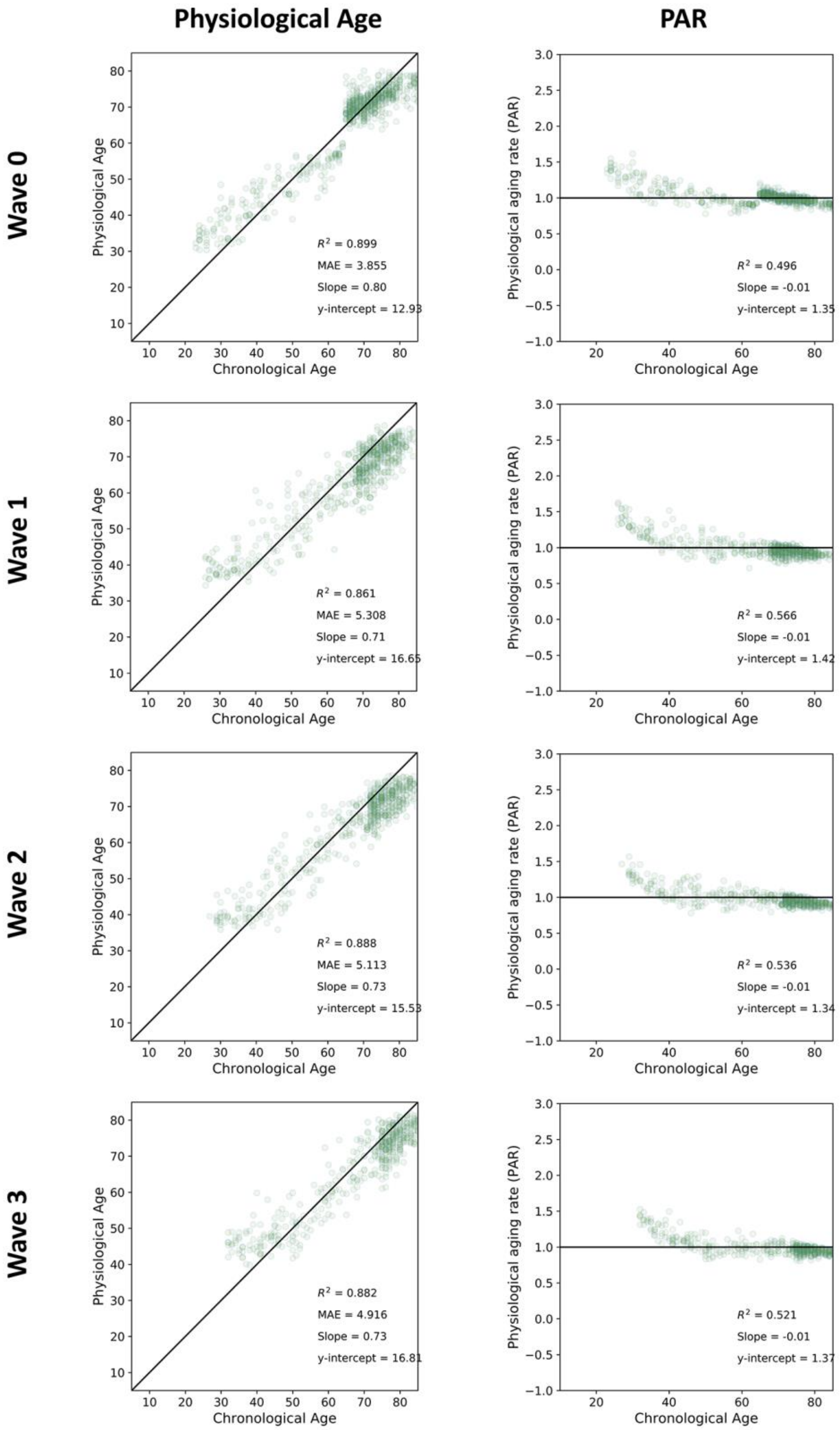


**Supplementary Figure 5. Plots of physiological age and PAR against chronological age for all follow-up studies of InCHIANTI using the full-trait RFC model.** Physiological wages were well-correlated to chronological age while PAR measurements were weakly correlated with chronological age across all follow-up studies.

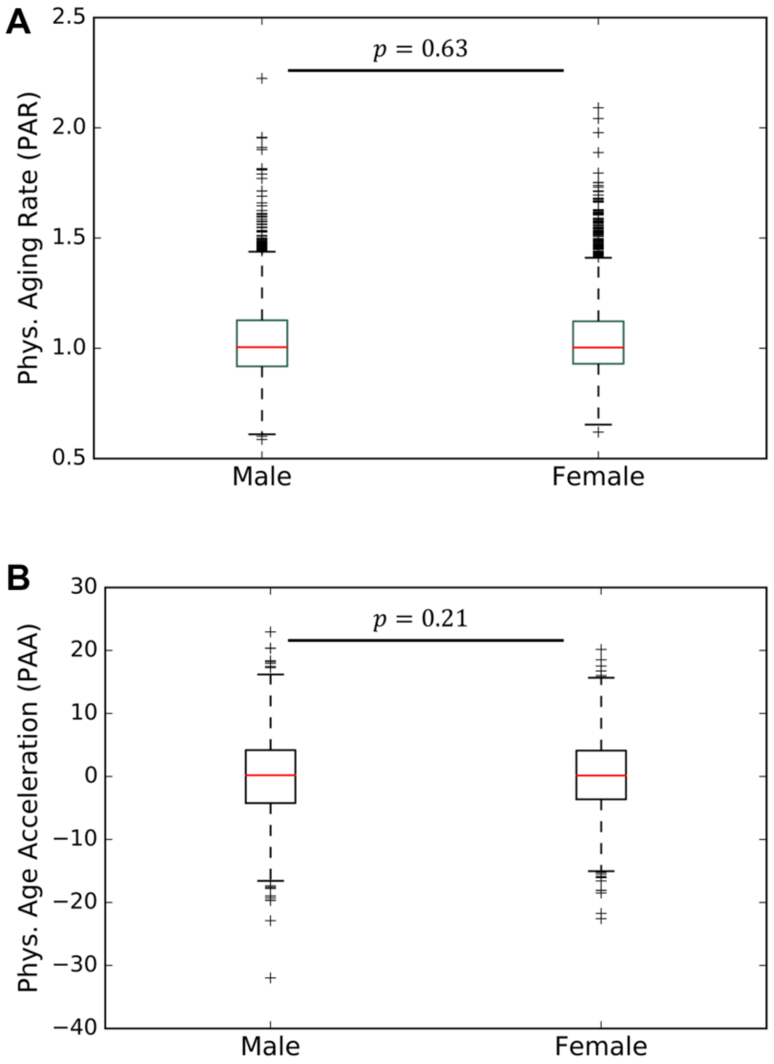


**Supplementary Figure 6. Gender-separated analysis.** (**A**) There is no significant difference between male and female participants in the physiological aging rate (PAR) obtained from the RFC model on the SardiNIA dataset. (**B**) Likewise, there is no significant difference in the physiological age acceleration measurements (PAA). See supplementary section on age acceleration calculation for a description of the PAA measurement. Reported *p*-values correspond to independent *t*-test of means.

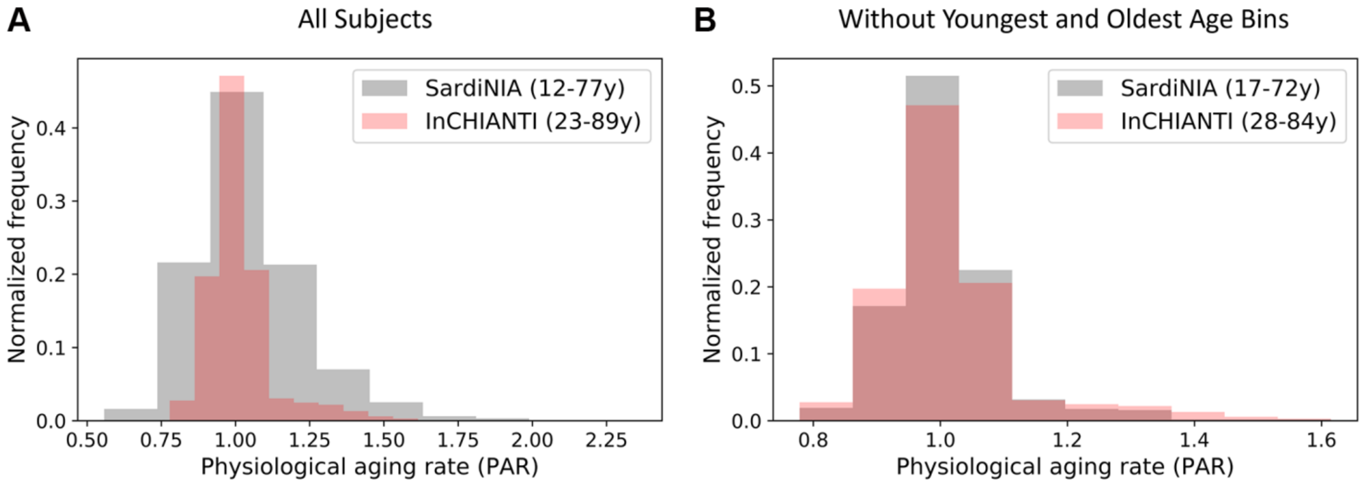


**Supplementary Figure 7.** Comparison of distribution of PARs in the SardiNIA (gray) and InCHIANTI (red) population studies; (**A**) for all individuals in each study; (**B**) for individuals that were not in the extreme-age (i.e. oldest and youngest) bins.

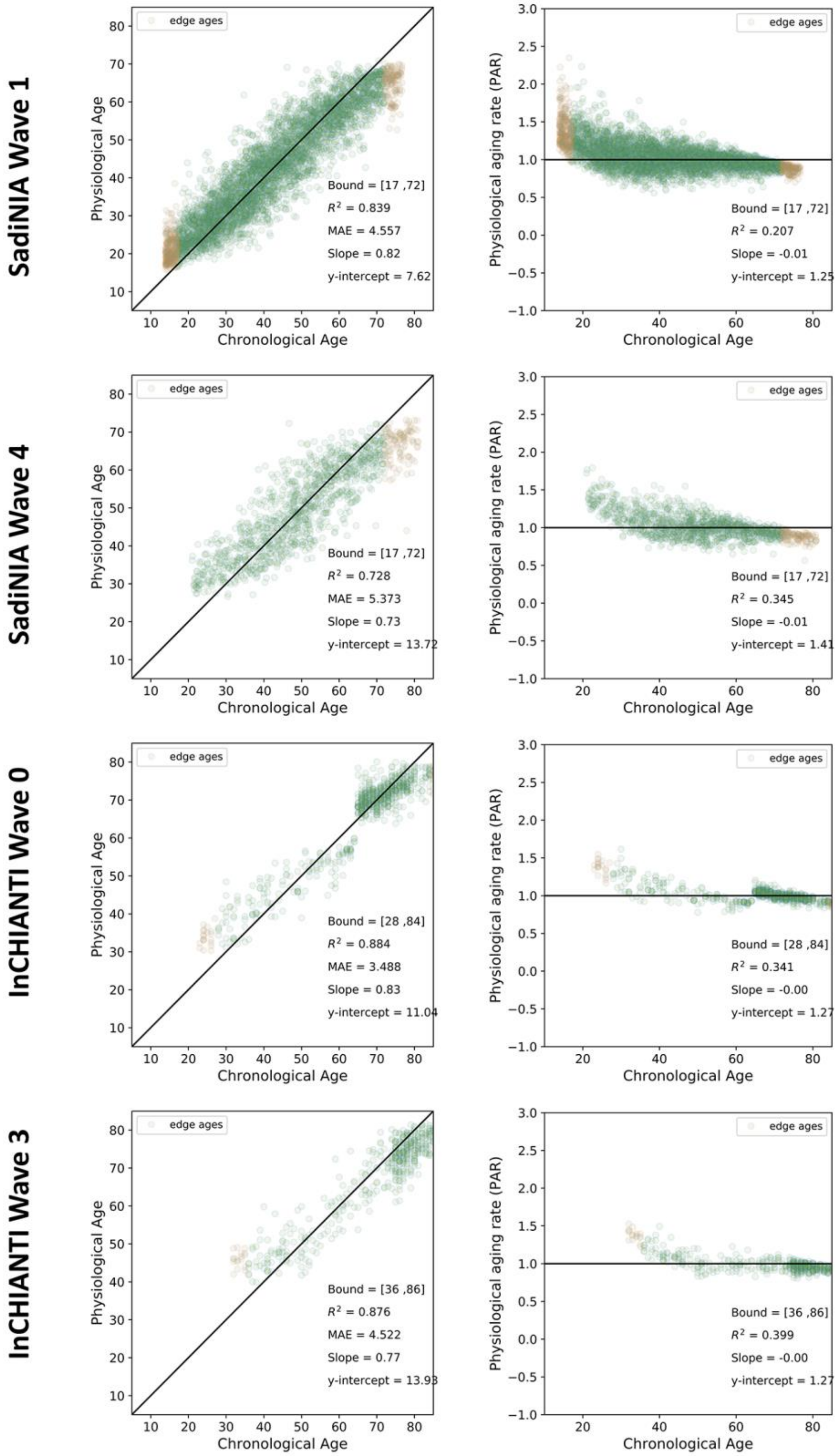


**Supplementary Figure 8. Removal of youngest and oldest age bins provides more balanced PAR distribution and produces similar results.** Shown are plots of physiological age and PAR against chronological age for the baseline and final follow-up studies of SardiNIA and InCHIANTI with the youngest and oldest age bins (colored brown) removed. “Bound” refers to the range of age bins that was used to compute the statistics (R2, MAE, slope and intercept of regression lines) shown in each plot.

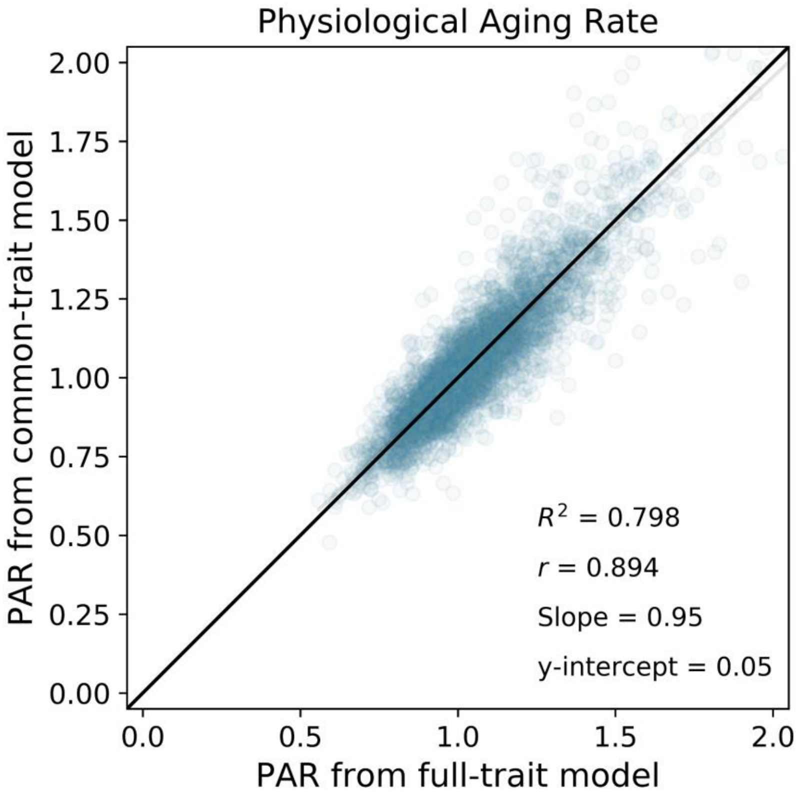


**Supplementary Figure 9. Common clinical and cardiovascular traits model.** Physiological aging rates (PAR) measured using the common-trait RFC model were highly correlated with the PAR measurements obtained from the full-trait RFC model. All figures were constructed using data from the baseline SardiNIA study.

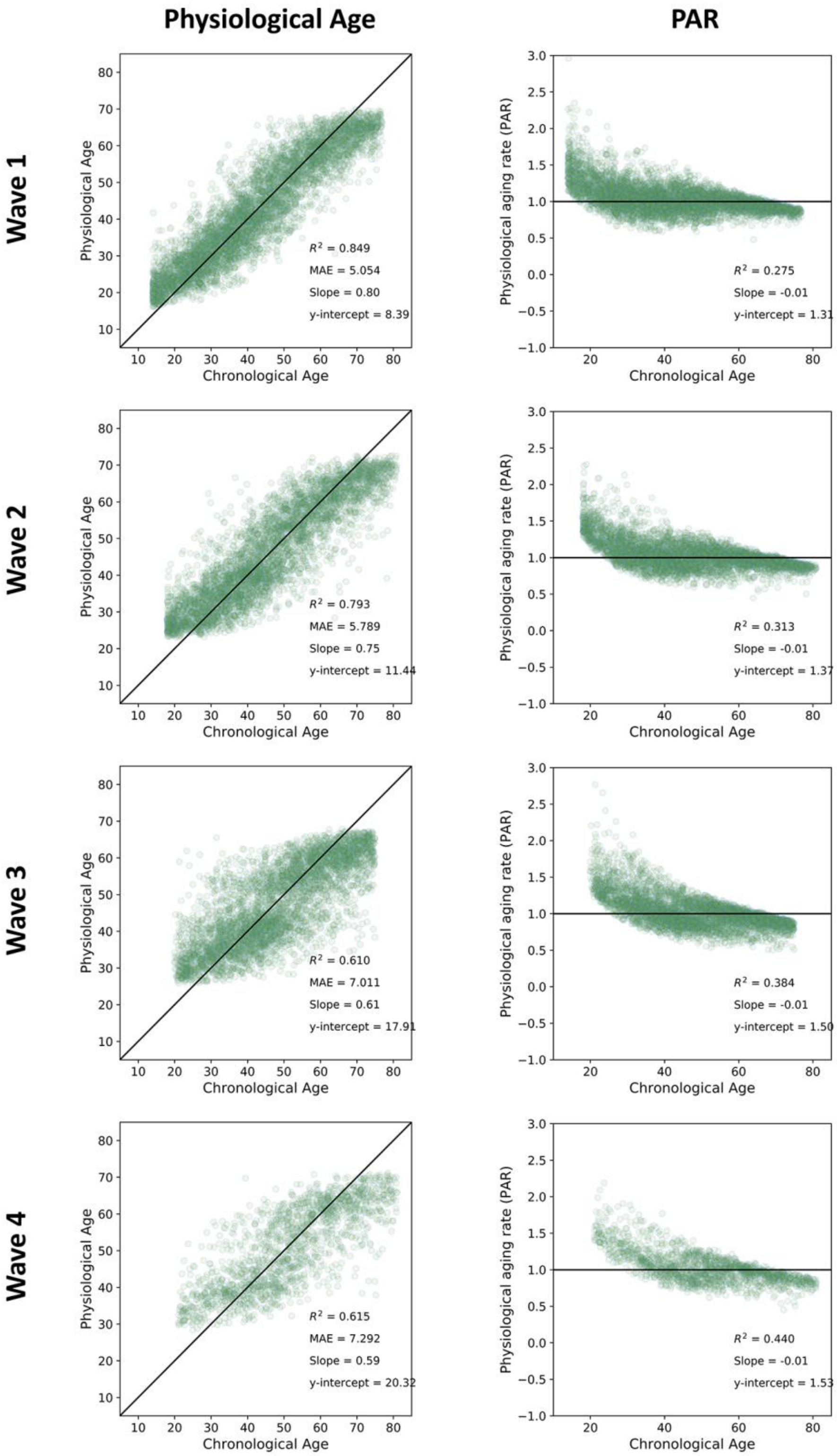


**Supplementary Figure 10. Plots of physiological age and PAR against chronological age for the baseline and follow-up studies of SardiNIA using the common-trait RFC model.** Results were comparable to the full-trait model. Physiological wages were well-correlated to chronological age while PAR measurements were weakly correlated with chronological age across all follow-up studies.

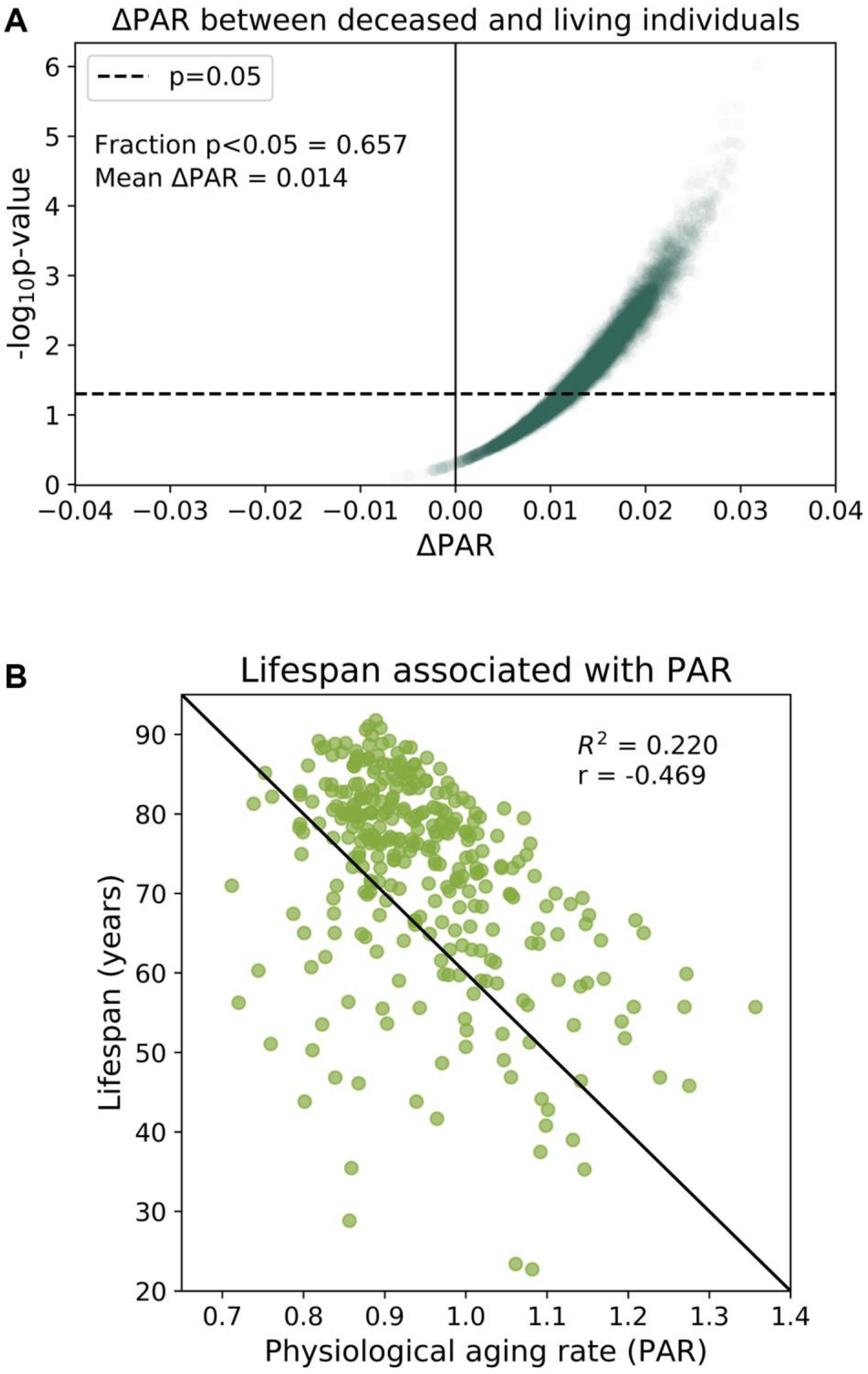


**Supplementary Figure 11. Physiological aging rates from the common-trait model are associated with mortality.** (**A**) 329 deceased participants were randomly paired with an age-matched living participant in the baseline SardiNIA study. We calculated the difference in the mean common-trait PAR measurements of the two groups, **Δ**PAR = $PAR_{deceased} - PAR_{living}$ and the corresponding *p*-value from a one-sided one-sample *t*-test for **Δ**PAR>0. The age-matched grouping was performed 10000 times and **Δ**PAR and *p*-values were calculated for each of the 10000 comparisons. The fraction of significantly greater than zero **Δ**PAR values ($p < 0.05$) was 65.7% and the mean **Δ**PAR was 0.014. Nearly all comparisons (>99%) had **Δ**PAR>0, which indicated that $PAR_{deceased} > PAR_{living}$ on average. (**B**) Lifespans for individuals living past 60 years were negatively correlated with PARs ($r = -0.469$).

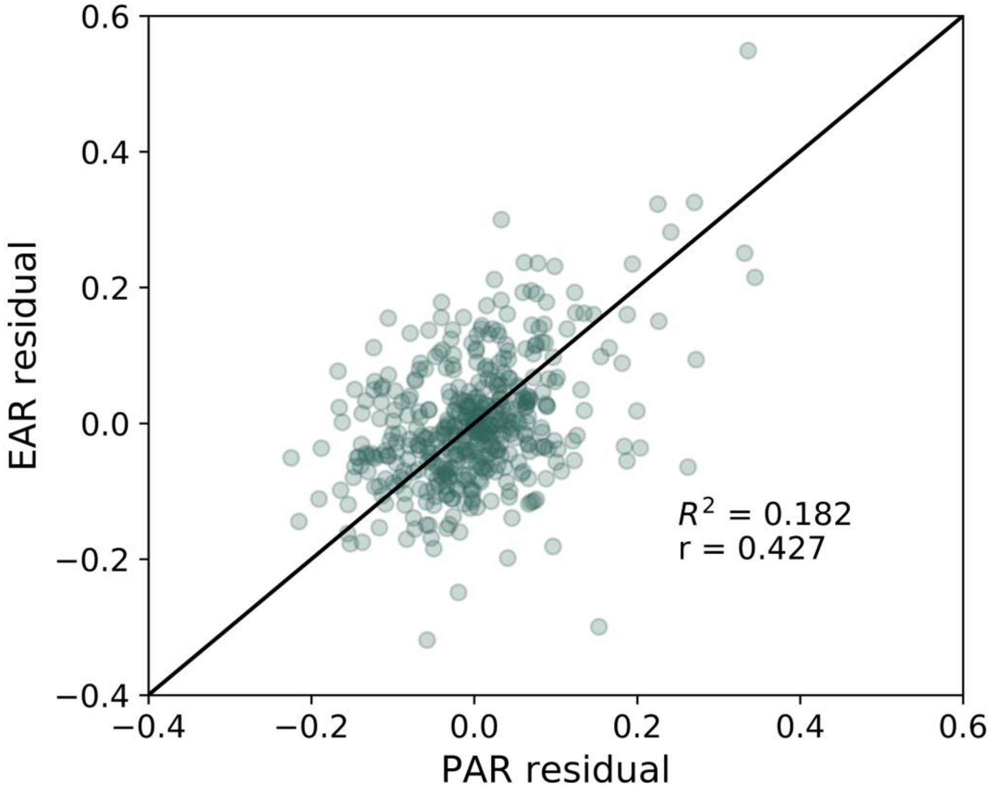


**Supplementary Figure 12. Correlation between epigenetic and physiological aging rate residuals in the InCHIANTI study.** Residuals (*x*) were calculated using an ordinary least-squares regression model: EAR or PAR = $\alpha_1$Age + $\alpha_2$Sex + *x*, which served to remove the effects of chronological age and sex from the aging rates. EAR and PAR measures were still positively correlated after this adjustment.

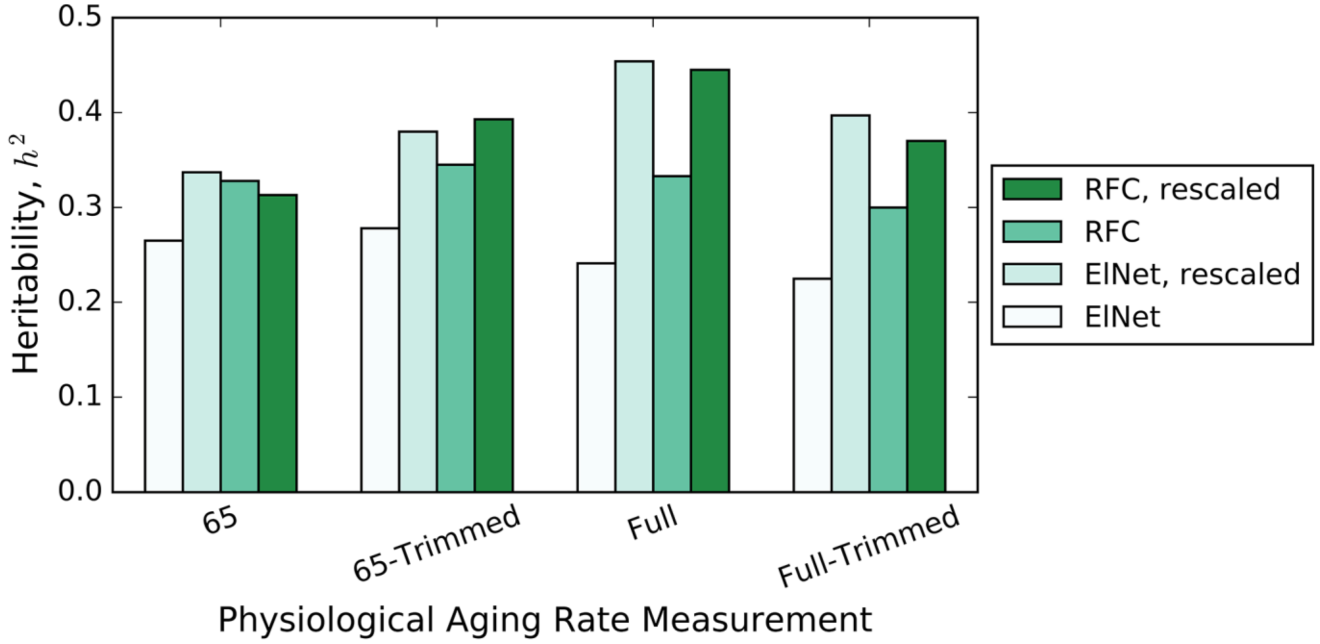


**Supplementary Figure 13. Heritability scores for PARs obtained from different models on the SardiNIA data.** Trimmed data refers to data where the oldest and youngest age bins were removed before model training and testing.

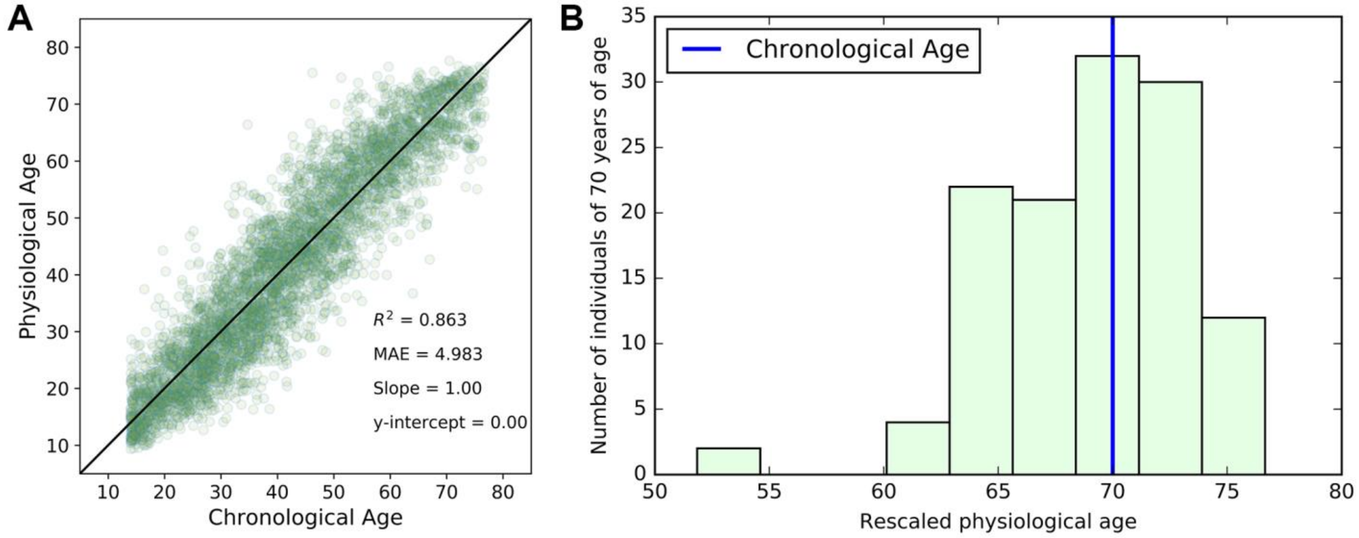


**Supplementary Figure 14. Linear rescaling of physiological ages.** (**A**) Linearly rescaling of the predicted physiological age reduced the age-dependent imbalance in PAR distributions and forced a regression slope of 1.00 between rescaled physiological age and chronological age. (**B**) Rescaling the physiological age preserved the assumption of approximately normally distributed physiological ages around the corresponding chronological age (blue line) for advanced ages (individual of 70 years of age are depicted).

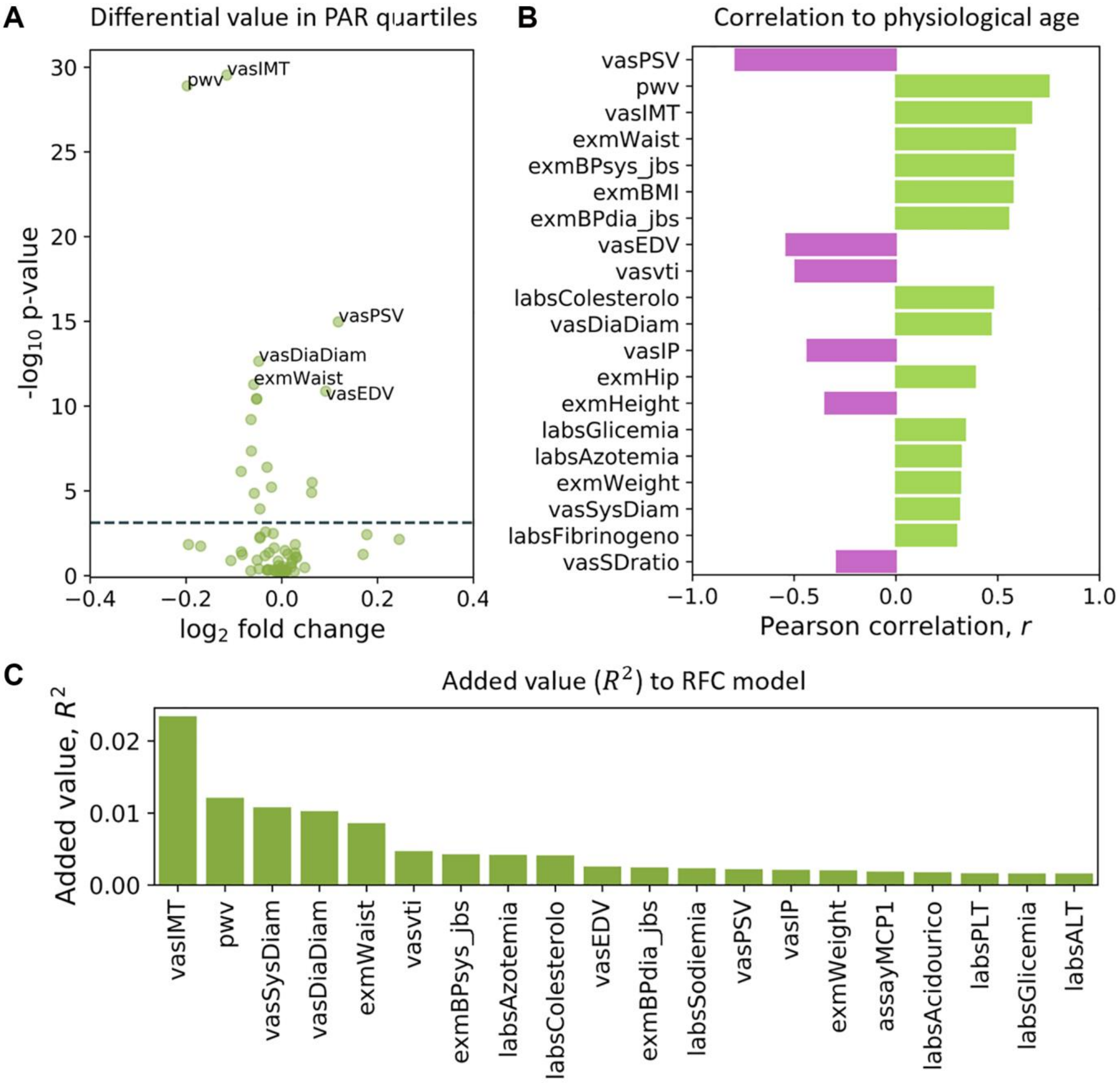


**Supplementary Figure 15. Top traits from common-trait model.** (**A**) Volcano plot of the top traits in the common-trait model included CCA intima media thickness (vasIMT), pulse wave velocity (pwv), peak systolic velocity (vasPSV), diastolic CCA diameter (vasDiaDiam), waist circumference (exmWaist), and end diastolic velocity (vasEDV). Significance was determined from a two-tailed students *t*-test on trait values from the top and bottom PAR quartiles. The dotted line corresponds to a Bonferroni-corrected *p*-value threshold of $p = 7.46 \times 10^{-4}$ calculated from single-test threshold of $p = 5.00 \times 10^{-2}$. The top traits were very similar for the full-trait model. (**B**) Traits rank-ordered by Pearson correlation r with physiological age measured using the common-trait RFC model on W1 data of SardiNIA. Top ranked traits were very similar to those from the full-trait model. (**C**) Traits rank-ordered by the approximate added predictive value measured using $R^2$ loss from $R^2 = 0.838$ for the common-trait RFC model in the baseline (W1) study of SardiNIA. $R^2$ added value was averaged over 500 training-testing iterates for each feature.

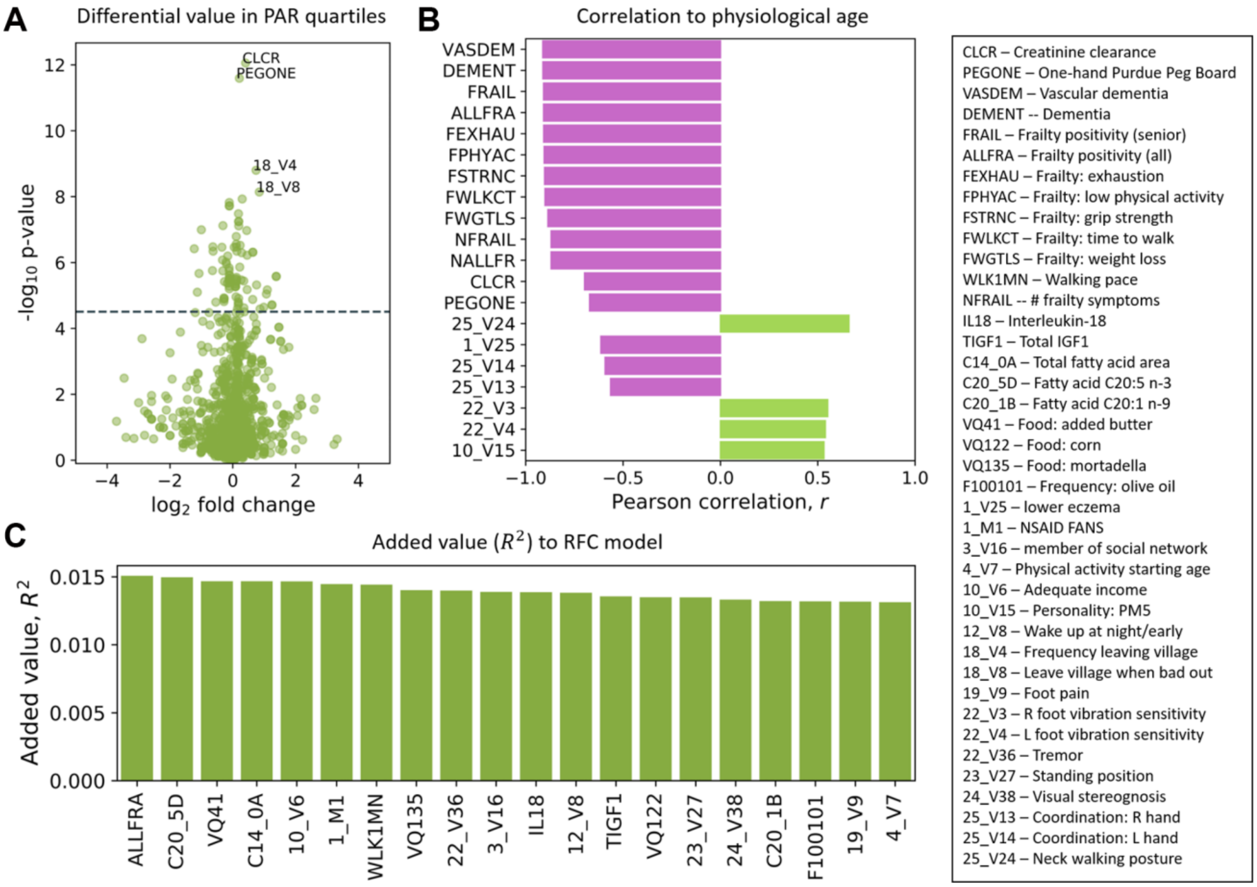


**Supplementary Figure 16. Top traits from InCHIANTI.** (**A**) Volcano plot of the top traits in the RFC model. Significance was determined from a two-tailed students *t*-test on trait values from the top and bottom PAR quartiles. The dotted line corresponds to a Bonferroni-corrected *p*-value threshold of $p = 3.12 \times 10^{-5}$ calculated from single-test threshold of $p = 5.00 \times 10^{-2}$. (**B**) Traits rank-ordered by Pearson correlation r with physiological age measured using the RFC model on W0 data of InCHIANTI. (**C**) Traits rank-ordered by the approximate added predictive value measured using $R^2$ loss from a baseline of $R^2 = 0.702$ for the RFC model (without feature selection) in the baseline (W0) study of InCHIANTI. $R^2$ added value was averaged over 500 training-testing iterates for each feature.

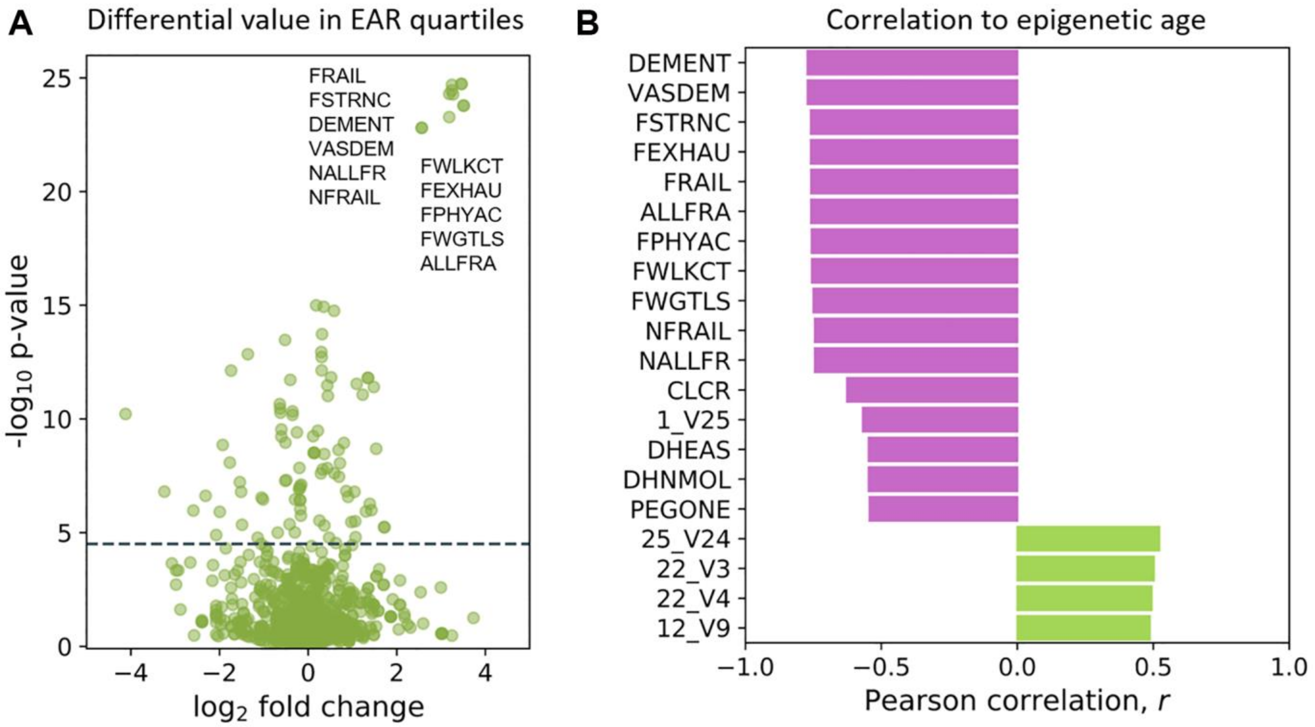


**Supplementary Figure 17. Top traits according to InCHIANTI epigenetic age and EAR.** (**A**) Volcano plot of the top traits for the epigenetic aging rate (EAR). Significance was determined from a two-tailed students *t*-test on trait values from the top and bottom EAR quartiles. The dotted line corresponds to a Bonferroni-corrected *p*-value threshold of $p = 3.12 \times 10^{-5}$ calculated from single-test threshold of $p = 5.00 \times 10^{-2}$. (**B**) Traits rank-ordered by Pearson correlation r with epigenetic age measured using the Horvath model on baseline InCHIANTI data. Trait rankings were highly similar to the rankings obtained using physiological age and PAR.

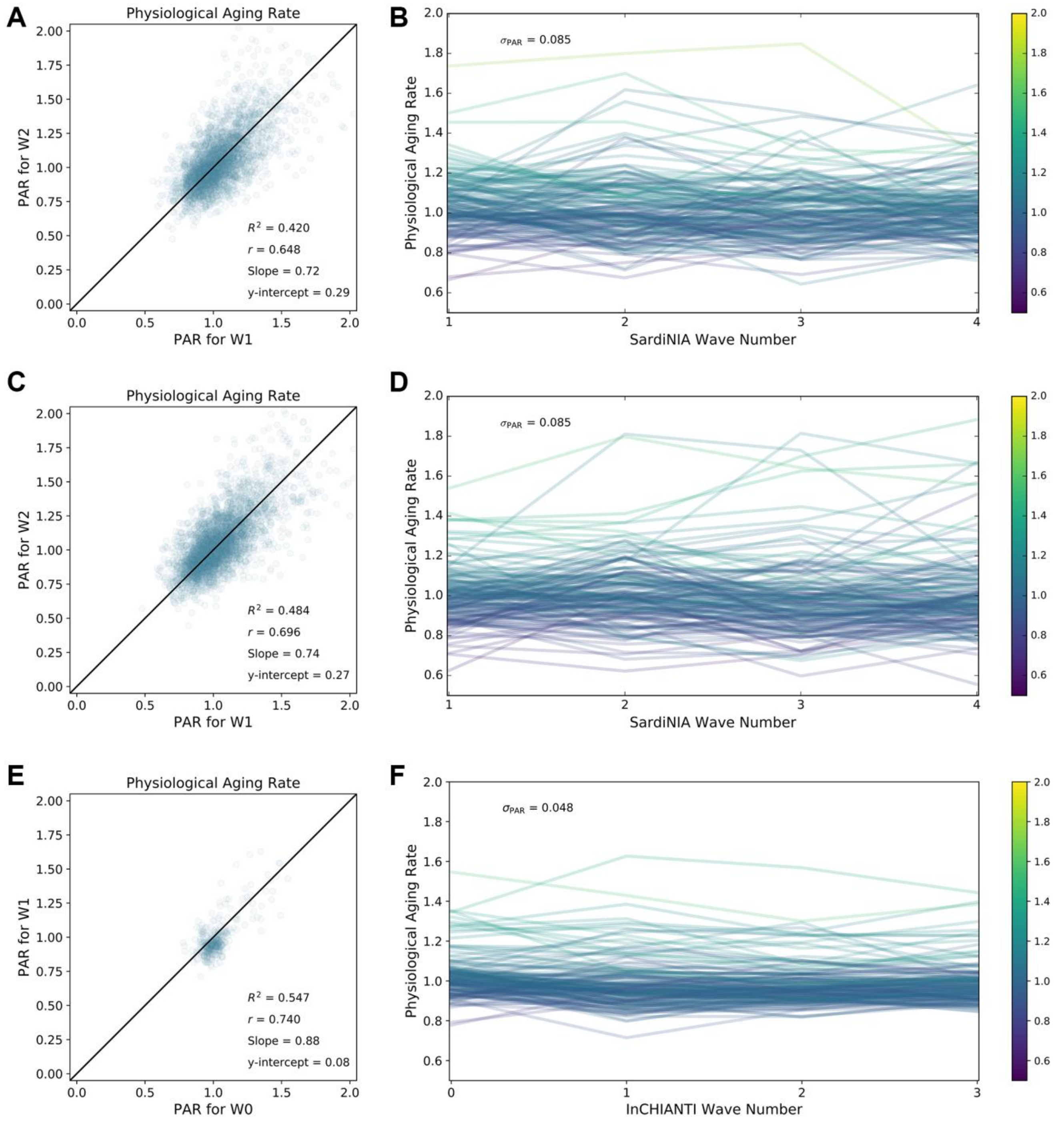


**Supplementary Figure 18. Physiological aging rates (PAR) were reproducible across follow-up studies.** (**A**) PAR values measured in the baseline (W1) study plotted against the PAR values measured in the first follow-up study (W2) of SardiNIA ($\Delta t \approx$ 3 years). PARs across W1 and W2 observed a Pearson correlation of r = 0.648. (**B**) Representative plot of PAR trajectories for 150 individuals across the four SardiNIA timepoints. Trajectories colors were mapped according to an individual's baseline PAR and showcased qualitative stability for a given individual with respect to others. (**C**–**D**) Corresponding plots for the common-trait model trained on SardiNIA data. (**E**–**F**) Corresponding plots for the full-trait RFC model trained on InCHIANTI data.

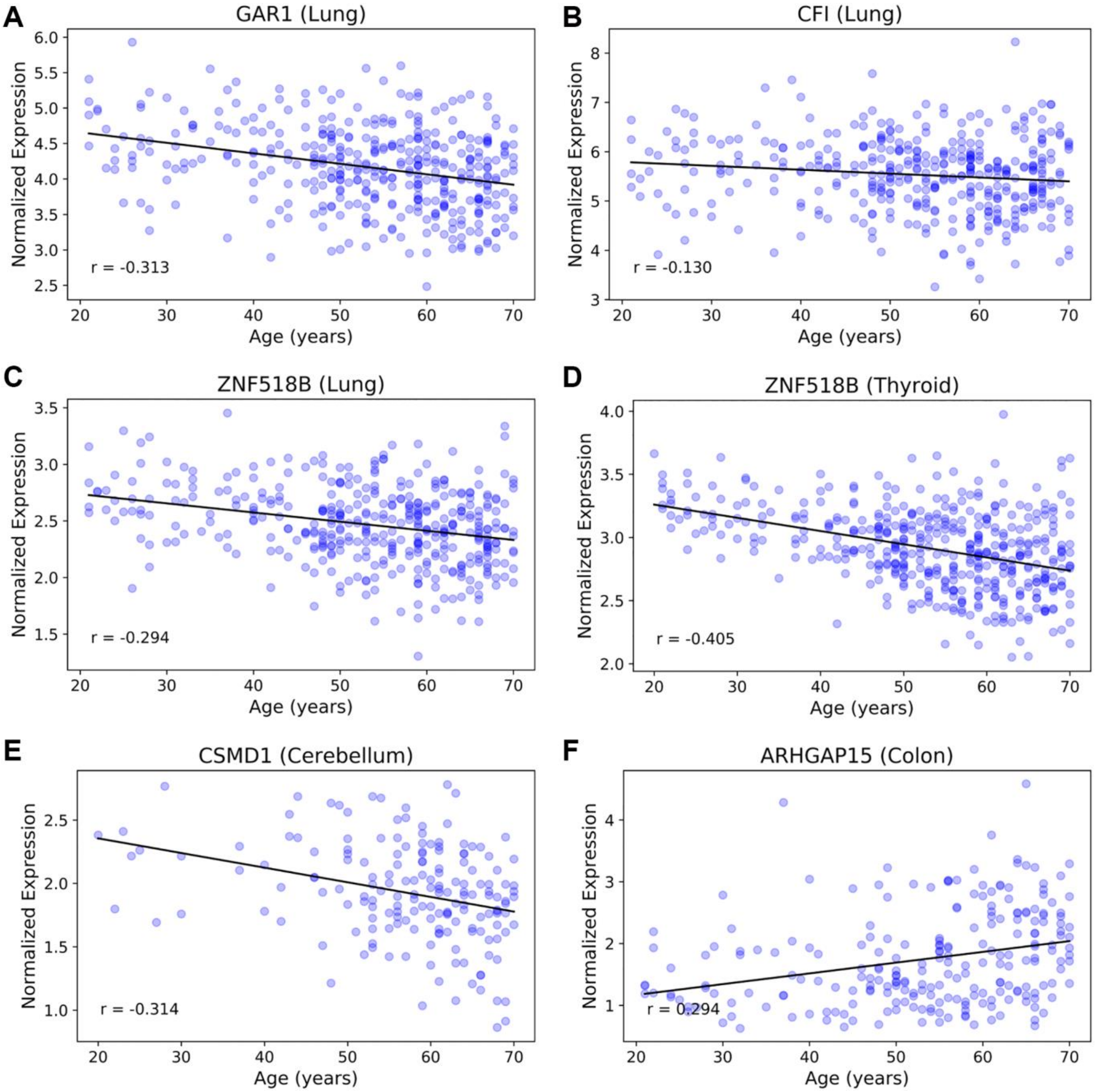


**Supplementary Figure 19. Gene expression values across age in GTEx human tissues for top genome-wide significant loci.** (**A**) Gar1 age-associated expression in GTEx lung tissue samples. (**B**) CFI age-associated expression in GTEx lung tissue samples. Gar1 was down-expressed in several tissues including heart (AA), lung, and colon while CFI did not appear to have strong age-dependent expression patterns in any tissues. (**C**) ZNF518B age-associated expression in GTEx lung tissue samples and (**D**) GTEx thyroid tissue samples. ZNF518B was down-expressed ($|r| > 0.2$) in the all non-brain GTEx tissues investigated: lung, thyroid, colon, heart (AA and LV), liver. (**E**) CSMD1 was greatly down-expressed ($r > -0.4$) with age in the cerebellum but not in non-brain tissues, which corroborates associations between CSMD1 and neurodegenerative disease and cognitive aging. (**F**) ARHGAP15 was up-expressed in colon only and is associated with diverticulitis and colorectal cancer.
