# Supplementary material for: Predicting physiological aging rates from a range of quantitative traits using machine learning
Source: Aging (Albany NY). 2021 Oct 29;13(20):23471–516. doi: 10.18632/aging.203660 (PMC8580337; doi:10.18632/aging.203660)
Supplement: Supplementary Table 1 [file aging-13-203660-s003.pdf]

SUPPLEMENTARY TABLE

Supplementary Table 1. Common clinical and cardiovascular traits included in the common-trait model for the SardiNIA study.

Trait	Units/Measurement	Description
labsRBC	10^6/uL	Red blood cell count
labsHB	g/dL	Hemoglobin lab test
labsMCV	fL	Mean corpuscular volume
labsMCH	pg	Mean corpuscular hemoglobin
labsWBC	10^3/uL	White blood cell count
labsPercNE	percentage	Neutrophil percentage
labsPercLY	percentage	Lymphocyte percentage
labsPercMO	percentage	Monocyte percentage
labsPercEO	percentage	Eosinophil percentage
labsPercBA	percentage	Basophils percentage
V1_V5		
labsPLT	10^3/uL	Platelet count
labsHBF	percentage	Fetal hemoglobin test
labsHBA2	percentage	Hemoglobin A2 test
labsG6PD	UI/dL	Glucose-6-phosphate dehydrogenase level
labsGlicemia	mg/dL	Glucose level
labsInsulinemia	mg/dL	Insulin level
labsAzotemia	mg/dL	Nitrogen level
labsALT	U/L	Alanine aminotransferase test
labsAST	U/L	Aspartate aminotransferase test
labsGammaGT	U/L	Gamma-glutamyltransferase test
labsFibrinogeno	mg/dL	Fibrinogen level
labsSideremia	mg/dL	Iron level
labsTransferrina	mg/dL	Transferrin level
labsBilirubinad	mg/dL	Fractionated bilirubin level
labsBilirubinat	mg/dL	Total bilirubin level
labsAcidourico	mg/dL	Uric acid level
labsSodiemia	mEq/L	Sodium level
labsPotassiemia	mEq/L	Potassium level
labsVES	mm/h	Erythrocyte sedimentation rate
labsPCR	mg/dL	C-reactive protein level
labsTSH	uU/L	Thyroid stimulating hormone level
labsFt4	mg/dL	Thyroid Ft4 level
assayAdip	mg/mL	Adiponectin level
assayLeptin	pg/mL	Leptin level
assayMCP1	pg/mL	Monocyte chemoattractant protein level
assayIL6		Interleukin-6 level

labsMCHC		
labsHtc		
labsMO_COUNT		
labsEO_COUNT		
labsBA_COUNT		
labsLY_COUNT		
labsNE_COUNT		
labsCreatinina	mg/dL	Serum creatinine level
labsColesterolo	mg/dL	Total cholesterol level
labsHDL	mg/dL	HDL cholesterol level
labsTrigliceridi	mg/dL	Triglycerides level
exmHeight	cm	Height
exmWeight	kg	Weight
exmWaist	cm	Waist circumference
exmHip	cm	Hip circumference
exmBMI	kg/m^2	Body mass index
exmBPsys_jbs		Supine blood pressure systolic
exmBPdia_jbs		Supine blood pressure diastolic
pwv	cm/s	Pulse wave velocity
vasPSV		Peak systolic velocity
vasEDV	mL	End diastolic velocity
vasIP		Pulsatility index
vasSDratio		Systolic-diastolic ratio
vasAT		Acceleration time
vasvti		Integral time velocity
vasSysDiam		Systolic CCA diameter
vasDiaDiam		Diastolic CCA diameter
vasIMT		CCA intima media thickness

Full trait descriptions for SardiNIA and InCHIANTI are available in the Supplementary Materials.
